# Decrease in UCP1 by sustained high lipid promotes NK cell necroptosis to exacerbate nonalcoholic liver fibrosis

**DOI:** 10.1038/s41419-024-06910-4

**Published:** 2024-07-20

**Authors:** Min Gu, Yu Zhang, Zhijie Lin, Xiangyu Hu, Yaqin Zhu, Weiming Xiao, Xiaoqin Jia, Weiwei Chen, Guotao Lu, Weijuan Gong

**Affiliations:** 1 Department of Gastroenterology, Affiliated Hospital of Yangzhou University, Yangzhou, PR China; 2 Department of Basic Medicine, School of Medicine, Yangzhou University, Yangzhou, PR China; 3 Jiangsu Key Laboratory of Integrated Traditional Chinese and Western Medicine for Prevention and Treatment of Senile Diseases, Yangzhou, PR China; 4 Jiangsu Key Laboratory of Zoonosis, Yangzhou, PR China

**Keywords:** Immune cell death, Mechanisms of disease

## Abstract

Uncoupling protein 1 (UCP1) catalyzes the leak of protons across the mitochondrial inner membrane for thermogenesis. Compromised NK cell activity is involved in the occurrence of nonalcoholic liver fibrosis. Here, decreased UCP1 in NK cells was identified in patients with advanced nonalcoholic fatty liver disease. Although no obvious changes were observed in the NK cells of physiologic UCP1^−/−^ mice (8–10 weeks old), impaired NK cell bioactivity was shown in methionine–choline-diet (MCD)-fed UCP1^−/−^ mice and involved in the acerbation of nonalcoholic steatohepatitis (NASH) progress to liver fibrosis. Moreover, UCP1-deficient NK cells were responsible for the aggravation of liver fibrosis, as confirmed in MCD-fed UCP1^flox/flox^-NCR1^cre^ mice. Acerbation of liver fibrosis was also seen in wild-type mice when their endogenous NK cells were replaced with UCP1^−/−^ NK cells. Transcriptions of mitophagy-associated molecules in UCP1^**−/−**^ NK cells were enhanced according to RNA-seq. Electron microscopic results showed mitochondrial injuries and autophagic vesicles in MCD-fed NK^WT^ cells, PA-treated NK^WT^ cells, or physiologic NK^KO^ cells. However, the co-existence of UCP1 deficiency and high lipid can synergistically induce NK cell necroptosis via DRP1^S616^ accompanied with reduced mitophagy. Finally, The UCP1 in NK cells was downregulated when treated by sustained high PA (600 μM) via the PPARγ/ATF2 axis. Thus, persistent high-lipid treatment not only decreases UCP1 expression but also combines with reduced UCP1 to promote NK cell necroptosis, and it is involved in NASH progression to fibrosis.

## Introduction

Nonalcoholic fatty liver diseases (NAFLDs) affect about 24% of the general population and up to 70% of overweight people. NAFLDs include hepatic steatosis (more than 5% of liver weight consisting of fat), nonalcoholic steatohepatitis (NASH), hepatic fibrosis, and hepatocellular carcinoma [1, 2]. A study showed that 44% of participants with nonalcoholic fatty liver (NAFL) developed NASH and 22% developed fibrosis [3]. The pathogenesis of NAFLD remains incompletely understood. Liver cells consist of hepatocytes (~78%) that exert liver primary function, such as lipid metabolism and bile secretion, and non-parenchymal cells, such as liver sinusoidal endothelial cells, Kupffer cells (KCs), hepatic stellate cells (HSCs), and hepatic NK cells. KCs play a key role in liver inflammation via production of inflammatory cytokines, including TNF-α, IL-1β, and IL-6 [4, 5]. In response to inflammation, HSCs are activated to secrete more collagen and cause fibrosis [6, 7]. Liver NK cells have dual effects on NALFD: they can amplify inflammatory responses at stage of NASH and inhibit liver fibrosis via inhibiting polarization of M2 KCs and killing activated HSCs [8–10].

Bioactivity of NK cell is modulated by its intrinsic metabolism [11]. In general, inactivated NK cells acquire energy by low-level mitochondrial oxidative phosphorylation (OXPHOS). However, activated NK cells increase glycolysis and OXPHOS to provide energy and ribose molecules for cell proliferation [12, 13]. The frequency and function of NK cells decrease in people with obesity [14, 15]. High free fatty acids (FFAs) promoted fatty acid oxidative phosphorylation in NK cells and inhibited NK cell function [16, 17]. Abnormal mitochondria also affected energy production and NK cell function. Fragmentation of the mitochondria led to NK cell inability against tumor [18].

Mitochondrial uncoupling proteins belong to the solute carrier SLC25 family. Uncoupling protein 1 (UCP1) is present in adipocytes, and it transports protons in the inner membrane of the mitochondria to mediate non-tremor thermogenesis [19, 20]. Given that UCP1-deficient cells are likely to enter a state of death after being exposed to reactive oxygen species (ROS), UCP1 can exert non-thermogenic activities, such as regulating oxidative stress [21]. Evidence showed that UCP1 can be transcribed in thymus tissue and thymocytes [22, 23]. The present study aimed to analyze how the loss of cell-intrinsic UCP1 affects NK cell bioactivity and the severity of nonalcoholic liver fibrosis, and how fatty acids regulate UCP1 expression in NK cells.

## Materials and methods

### Human patients

Peripheral blood of patients with NAFLD was collected from the Affiliated Hospital of Yangzhou University. Patients with other liver diseases or a history of alcohol consumption were excluded. Demographic and clinical data were collected, as shown in Supplementary Table 1.

### Animal model

UCP1-knockout mice (strain no. T037633), *ucp1*^flox/flox^ mice (strain no. T013232), and *NCR1*^cre^ mice (strain no. T005674) were purchased from GemPharmatech (Nanjing, China). C57BL/6 wild-type (WT) mice were provided by the Comparative Medical Center of Yangzhou University (Yangzhou, China). The *ucp1*^flox/flox^ mice were crossed with *NCR1*^Cre^ mice to obtain *ucp1*^flox/flox^*NCR1*^cre^ conditional knockout (cKO) mice. For methionine–choline-diet (MCD)-induced NAFLD, which was characterized by hepatic steatosis, inflammation, and fibrosis, mice (~8 weeks) were fed with MCD for 4 weeks [24]. Mice were housed at a constant temperature (25 ± 2 °C) and humidity (40%–60%), with a 12-h light and 12-h dark cycle. Appropriate food and water were also provided.

### Reagents and antibodies

The following reagents were used in this study: recombinant IL-2, IL-15 (Biolegend, San Diego, CA, USA), poly I:C (Absin, Shanghai, China), and palmitic acid (PA, Sigma, Darmstadt, Germany). The mouse antibodies used in flow cytometry included CD3 (17A2), NK1.1 (PK136), CD69 (H1.2F3), NKG2D (A10), CD36 (HM36), CD107a (1D4B), IFN-γ (XMG1.2), GZMB (NGZB), CD11b (M1/70), F4/80 (BM8), CD86 (GL-1), CD206(C068C2), and TNF-α (MP6-XT22). Human antibodies included CD3 (OKT3), CD56 (5.1H11), NKG2D (1D11), NKp46 (9E2), IFN-γ (4S.B3), and GZMB (QA16A02). All antibodies were obtained from BioLegend (San Diego, CA, USA) or Thermo Fisher (Waltham, MA, USA).

The antibodies used in western blot included Glut1 (E4S6I), HK2 (C64G5), ACC1 (C64G5), p-ACC1 (Ser79), CPT1a (8F6AE9), PI3K p110α (C73F8), mTOR (7C10), p-mTOR (Ser2448) (D9C2), Akt (11E7), p-Akt (Ser473) (D9E), c-Myc (D84C12), p-c-Myc (Ser62) (E1J4K), UCP1 (E9Z2V), AMPK (D63G4), p-AMPK (Thr172, D4D6D), PTEN (D4.3), PPARγ (81B8), p-PPARγ (Ser112) (Absin, Shanghai, China), PINK1 (ab23707), Parkin (Prk8), SQSTM1/p62 (D6M5X), LC3A/B (D3U4C), FUNDC1 (E2F4T), PGAM5 (E8C3L), NF-κB p65 (D14E12), p-NF-κB p65 (Ser536) (93H1), DRP1 (D6C7), p-DRP1 (Ser616) (D9A1), NLRP3 (D4D8T), caspase-1 (E9R2D), cleaved caspase-1 (Asp296) (E2G2I), GSDMD (E9S1X), cleaved GSDMD (Asp276) (E3E3P), p38 MAPK (D13E1e), p-p38 MAPK (Thr180/Tyr182, D3F9), ATF2 (D4L2X), RIP1 (D94C12), p-RIP1 (Ser166) (D8I3A), RIP3 (D4G2A), p-RIP3 (Ser227) (D6W2T), MLKL (D6W1K), and p-MLKL (Ser358, E7G7P), GPX4 (E5Y8K), ferritin (EPR3004Y), ALOX12 (E3O9P), TFAM (RM1035), PGC1a (EPR25162-281), NRF1 (EPR5554(N)), NRF2 (D1Z9C), iNOS (D6B6S), arginase-1 (D4E3M™), Stat1 (D1K9Y), phospho-Stat1 (Tyr701) (58D6), Stat6 (D3H4), phospho-Stat6 (Tyr641) (D8S9Y), caspase-3 (D3R6Y), cleaved caspase-3 (Asp175) (5A1E), caspase-8 (D35G2), cleaved caspase-8 (Asp387) (D5B2), NLRP3 (D4D8T), gasdermin D (E9S1X), cleaved gasdermin D (Asp276) (E3E3P), Bcl-2 (D17C4). All antibodies were obtained from Cell Signaling Technology (Boston, MA, USA) or Abcam (Cambridge, UK).

### Extracellular acidification rate

(ECAR)XFe-96 Extracellular Flux Analyzers (Seahorse Bioscience, MA, USA) were utilized to quantify the extracellular acidification rate (ECAR). NK cells were isolated and seeded at a density of 2 × 10^5^ cells/well onto 96-well Seahorse plates precoated with CellTak (BD Pharmingen). The plates were then centrifuged at 200 × *g* for 1 min and transferred to a CO_2_-free incubator set at 37 °C for 25 min to ensure optimal cell adhesion. The cellular measurements were conducted in Seahorse medium (Agilent, CA, USA) supplemented with 10 mM glucose, 2 mM glutamine, and 1 mM pyruvate (Sigma, Darmstadt, Germany). Sequential treatment with 10 mM glucose, 1 μM oligomycin, and 100 mM 2-DG was administered to evaluate glycolytic activity. Glycolytic capacity were calculated as per standard reference.

### Histology

Mouse livers were freshly isolated and fixed in 4% polyformaldehyde, embedded in paraffin, and cut into 5 μm sections. The sections were routinely stained by H&E, Masson’s trichrome, Sirius red, or Oil red.

#### H&E stain

The sections were then deparaffinized in xylene, hydrated in graded ethanol, and stained with hematoxylin for 5–10 min, followed by eosin staining for 30 s. The sections were then dehydrated in ethanol solutions, cleared in xylene, and mounted with permanent mounting medium.

#### Fibrosis stain

Masson’s trichrome staining (Jiancheng Company, Nanjing, China) and Sirius red staining (Solarbio, Shanghai, China) were performed using a commercially available kit. The sections were deparaffinized in xylene, rehydrated in graded ethanol, and stained using the kit according to the manufacturer’s instructions. The slides were then washed, dehydrated, and mounted for analysis.

#### Oil red O stain

Liver were embedded in OCT compound and frozen at −80 °C. The frozen liver were sectioned at a thickness of 10 μm using a cryostat microtome maintained at −20 °C. The slides were briefly thawed at room temperature and staining using the kit (Jiancheng, Nanjing, China) according to the manufacturer’s instructions.

### Flow cytometry

Single cells were collected and stained with fluorescently conjugated antibodies for 30 min at 4 °C. After being washed, the cells were resuspended in PBS and analyzed by flow cytometry using FACSVerse (BD Biosciences, NJ, USA) and FlowJo software for parameters.

### Detection of aspartate transaminase (AST), alanine transaminase (ALT), triglyceride (TG), cholesterol (CHO), and free fatty acids (FFAs)

All parameters were measured using commercial enzymatic assay kits in accordance with the manufacturer’s protocols. The detection kits for ALT, AST, TG, and CHO were purchased from Jiancheng Company (Nanjing, China). The kit for FFAs was supplied by Solarbio Company (Shanghai, China).

#### ALT (Jiancheng, Nanjing, China)

Under the conditions of 37 °C and pH 7.4, alanine aminotransferase (ALT) acted on the substrate composed of alanine and α-ketoglutarate, generating pyruvate and glutamate. After a fixed time of 30 min, 2,4-dinitrophenylhydrazine (DNPH) hydrochloride solution was added to terminate the reaction, and DNPH reacted with the carbonyl group in the ketone to form 2,4-dinitrophenylhydrazone. The hydrazone turned reddish-brown under alkaline conditions, and the enzyme activity was calculated based on the absorbance measured at 505 nm.

#### AST (Jiancheng, Nanjing, China)

Aspartate aminotransferase (AST) catalyzed the interconversion between α-ketoglutarate and aspartate to produce glutamate and oxaloacetate. Oxaloacetate could decarboxylate spontaneously to form pyruvate. Pyruvate reacted with 2,4-dinitrophenylhydrazine to produce 2,4-dinitrophenylhydrazone, which appeared reddish-brown in alkaline solution. The enzyme activity units of AST could be calculated using a standard curve based on the colorimetric method, thereby determining the enzyme activity of AST.

#### Triglycerides (TG) (Jiancheng, Nanjing, China)

Serum samples were incubated with reagents containing lipase and glycerol kinase, which hydrolyze triglycerides to glycerol and fatty acids. Glycerol then reacted with an enzyme and a chromogenic substrate, producing a colorimetric signal that was measured spectrophotometrically.

#### Cholesterol (CHO) (Jiancheng, Nanjing, China)

Serum samples were treated with a reagent containing cholesterol esterase and cholesterol oxidase, which hydrolyze cholesterol esters to cholesterol and hydrogen peroxide. The hydrogen peroxide then reacted with a chromogenic substrate, producing a colorimetric signal that was measured spectrophotometrically.

#### Free fatty acids (FFAs) (Solarbio, Shanghai, China)

FFAs were present in serum samples, and they formed copper complexes and dissolved in chloroform. Copper ion content was determined by the copper reagent method.

### NK cell sorting

Nonalcoholic fatty liver disease (NAFLD) peripheral blood samples were collected. PBMCs were isolated from the blood using density gradient centrifugation with Lymphocyte Separation Media (Multi Sciences, Hangzhou, China). Briefly, blood was diluted 1:1 with phosphate-buffered saline (PBS) and layered over lymphocyte separation media in a 2:1 v/v ratio. The sample was centrifuged at 500 × *g* for 20 min at room temperature, and the ring of PBMCs at the interface was collected. The collected PBMCs were then washed twice with PBS by centrifugation at 500 × *g* for 10 min at 4 °C. The cell pellet was resuspended in PBS for further downstream applications. Isolation of human NK cells was performed using a commercial kit for human NK cell separation (Miltenyi Biotec, Germany), and the cells were enriched by negative selection according to the manufacturer’s instructions.

#### Isolation of mouse NK cells

Single-cell suspensions were prepared from mouse spleens. CD49b magnetic beads (Miltenyi Biotec, Germany) were used to incubate with the single-cell suspensions at 4 °C for 15 min. The cells were washed twice with PBS buffer and then centrifuged at 500 × *g* for 10 min. The cells were resuspended in PBS buffer and subjected to positive selection using an LS magnetic column to isolate and purify NK cells.

### NK cell depletion and adoptive transfer

NK cells were depleted by injection of 400 μg of anti-NK1.1 (PK136, Bio X Cell) on days −3, −1, and +1 relative to MCD feeding. Isolation of NK cells from spleens of WT and KO mice, followed by collection and injection of NK cells (~1 × 106) via tail vein into recipient mice during MCD feeding. Injections were administered twice weekly for a total of 4 weeks.

### Rescued expression of UCP1

UCP1 lentiviral was purchased from Genechem (Wuhan, China). NK cells isolated from the spleens of UCP1-KO mice. NK cells were infected with lentiviruses together with polybrene (10 µg/ml) for 24 h. After 24 h, the transfected NK cells (~1 × 106) were collected and injected into UCP1-KO mice via the tail vein twice a week for 4-week period during the MCD model.

### Electron microscopy

NK cells were fixed with 2.5% glutaraldehyde and 1% osmic acid in 0.1 M PBS (pH 7.2) at 4 °C for 2 h and then dehydrated using a graded ethanol series. Samples were then embedded in Epon 812 resin and sectioned with a diamond knife on an ultramicrotome. The ultrathin sections were collected on copper grids and stained with 2% uranyl acetate and 0.1% lead citrate. A transmission electron microscope (HT7800, Yangzhou, China) was used to examine the morphologies.

### Lactic dehydrogenase (LDH) release

NK cells were treated with PA (dissolved in 10% BSA) or PBS solution. After 24 h, cell culture medium was collected and measured using a commercial LDH assay kit (Beyotime Biotechnology, Shanghai, China). The absorbance of the colored product was quantified using a plate reader, and the amount of LDH release was calculated on the basis of absorbance.

### Western blot

The proteins of cell lysis were subjected to SDS–PAGE and transferred to a PVDF membrane. After being blocked by 5% BSA, the membrane was incubated with primary antibody, followed by secondary antibody conjugated with horseradish peroxidase. The protein bands were then visualized by an imaging system.

### Statistics

All data were presented as mean ± SD. One- or two-way ANOVA was used to determine statistical significance among multiple groups. Two-tailed Student’s *t*-test was used for comparisons between two groups. Statistical analysis was conducted using GraphPad Prism 9. Statistical significance was indicated as *P < 0.05, **P < 0.01, and ***P < 0.001; ****P < 0.0001.

## Results

### Decreased UCP1 compromises NK cell function in individuals with NAFLD

**Fig. 1:**
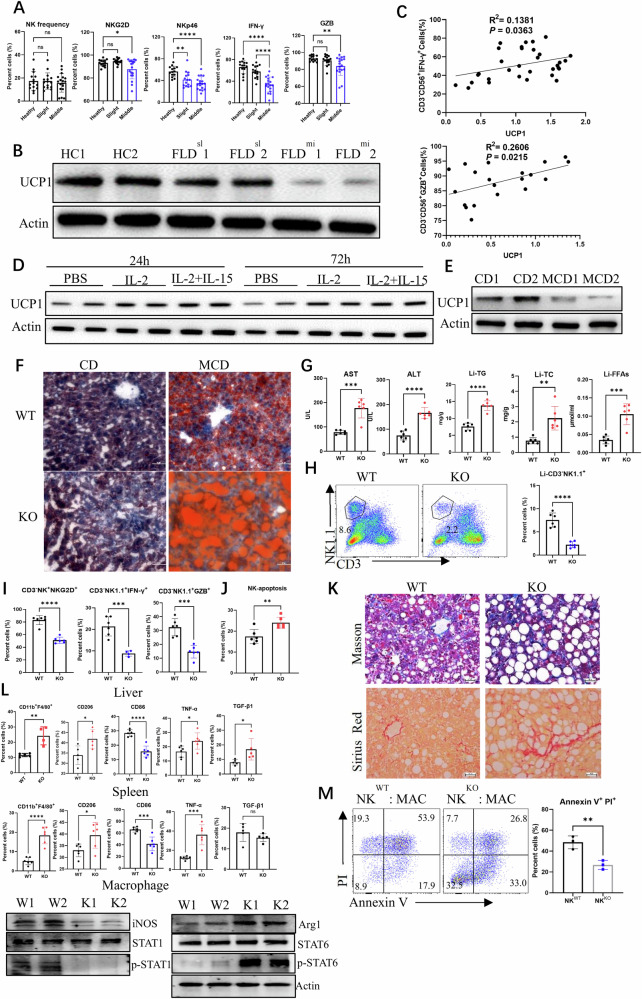
Compromised NK cell activities by decreased UCP1 in NAFLD individuals. **A** NK cell frequency and phenotype in slight or moderate NAFLD patients. **B** UCP1 levels in peripheral NK cells. **C** Correlations of UCP1 levels with IFN-γ and GZB productions of NK cells. **D** UCP1 of NK cells stimulated with IL-2 and IL-15 ex vivo. **E** UCP1 of NK cells from MCD-fed mice. **F** Liver droplets stained by oil red. **G** Serum AST and ALT, and liver TG, TC and FFAs. **H** Detection of hepatic NK cells. NKG2D, IFN-γ, GZB (**I**) of hepatic NK cells. **J** NK cell apoptosis detected by flow cytometry. **K** Liver sections stained by Masson and Sirius red. **L** Variations of macrophages in liver and spleen. **M** NK cell cytotoxicity against macrophages. Human NK cells were isolated from peripheral blood, and mouse NK cells were isolated from spleen. All animal and cell experiments were repeated at least twice. Ns no significance; *P < 0.05; **P < 0.01; ***P < 0.001; ****P < 0.0001.

Variations in peripheral NK cell (CD3^−^ CD56^+^) bioactivities were first investigated in patients with NAFLD at different stages (slight and middle) on the basis of ultrasonic diagnosis. The patients with middle NAFLD had remarkably decreased NKp46^+^, NKG2D^+^, IFN-γ^+^, and GZB^+^ NK cells in the peripheral blood, demonstrating the compromised bioactivity of NK cells in individuals with fatty livers (Fig. 1A). Simultaneously, the NAFLD^slight^ patient-derived NK cells had decreased UCP1 expression, whereas the NAFLD^middle^ patient-derived NK cells demonstrated considerable loss of UCP1 expression (Fig. 1B). The UCP1 level of NK cells examined by western blot was positively correlated with their production of IFN-γ and GZB (Fig. 1C). When the peripheral NK cells were stimulated with rIL-2 or the combination of rIL-2 and rIL-15 ex vivo for 24 or 72 h, UCP1 expression was upregulated (Fig. 1D). In mice with MCD-induced NAFLD, the splenic NK cells substantially decreased UCP1 expression (Fig. 1E). These results demonstrated that decrease in UCP1 in association with compromised NK cell bioactivity was involved in the pathogenesis of NAFLD.

Next, UCP1^−/−^ mice (Supplementary Fig. 1A) were used to analyze how UCP1 loss affected NK cell bioactivity and then the progression of NAFLD. No obvious changes in CD3^−^ NK1.1^+^ and CD3^−^ NK1.1^+^ NKG2D^+^ cell frequencies were observed in the spleen, blood, liver, and thymus of physiologic UCP1^−/−^ mice (Supplementary Fig. 1B, C). Under physiological state, the NK cell subsets (CD11b^+^ CD27^−^), apoptosis, and production of IFN-γ did not vary substantially (Supplementary Fig. 1D–F) in young KO mice (8–12 weeks old). These mice also had no increase in serum CHO, TG, FFAs, ALT, and AST (Supplementary Fig. 1G) at this time. However, exacerbated NAFLD in UCP1^−/−^ mice fed with MCD was identified by increased lipid accumulation in the liver (Fig. 1F); increased serum AST and ALT as markers for NASH; and elevated levels of TG, total CHO (TC), and FFAs in the liver (Fig. 1G). These UCP1^−/−^ mice demonstrated a decrease in NK cells in the liver (Fig. 1H and Supplementary Fig. 1H) and spleen (Supplementary Fig. 1I) after being fed with MCD. The UCP1^−/−^ NK cells in the liver also exhibited a decrease in the production of NKG2D, IFN-γ, and GZB (Fig. 1I) and increased apoptosis (Fig. 1J). Simultaneously, NKG2D, IFN-γ, and GZB were downregulated in splenic UCP1^−/−^ NK cells (Supplementary Fig. 1K). Meanwhile, the liver CD4^+^ and CD8^+^ T cells did not vary substantially (Supplementary Fig. 1J), whereas increased CD4^+^ and CD8^+^ T cells were seen in the spleen of MCD-fed UCP1^−/−^ mice (Supplementary Fig. 1L).

Severe liver fibrosis was observed in MCD-fed KO mice by Masson or Sirius red staining (Fig. 1K). Considering hepatic macrophages play key roles in the development of fibrosis [25], more M2-like macrophages with increased CD206, TNF-α, and TGF-β1 and decreased CD86 were present in the liver of MCD-fed KO mice, as compared with WT mice. A similar trend was found in the splenic macrophages of MCD-fed KO mice. M1 macrophage effector molecules including iNOS and p-STAT1 decreased, while ARG1 and p-STAT6 as M2 macrophage effector molecules increased (Fig. 1L). NK cells can maintain local homeostasis via killing inflammatory macrophages [26]. The present study showed that the NK cells of MCD-fed UCP1^−/−^ mice cannot efficiently exert cytotoxicity against macrophages (Fig. 1M), confirming the downregulation of NK cell bioactivity. In summary, compromised NK cell activity with decreased UCP1 was involved in the progression of NAFLD, particularly in liver fibrosis.

### NK cell-intrinsic deficiency of UCP1 facilitates NASH progression to fibrosis

**Fig. 2:**
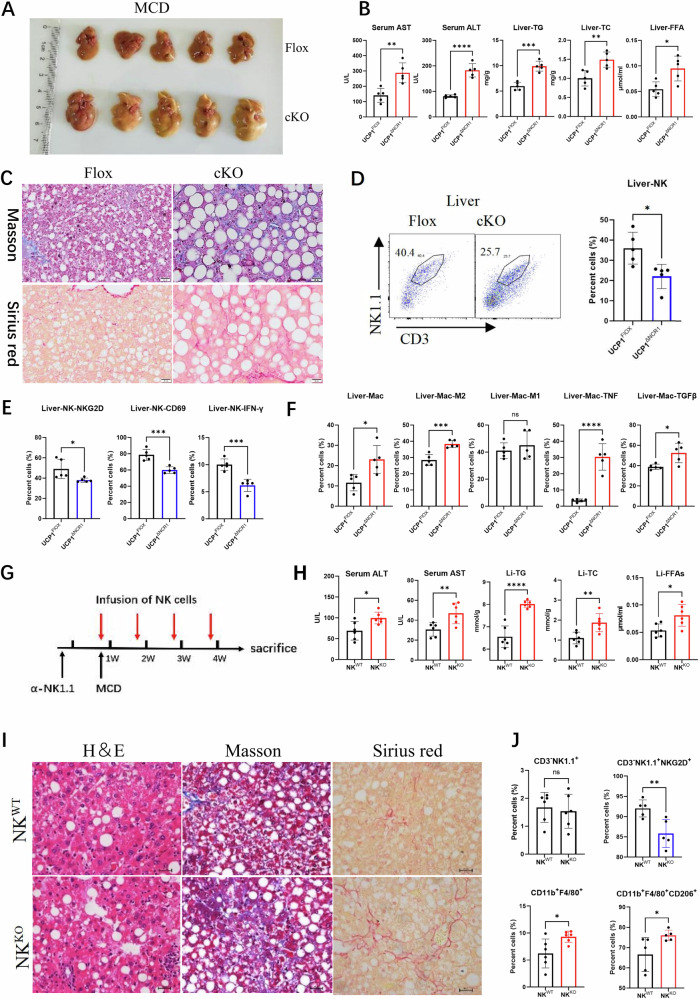
Intrinsic deficiency of UCP1 in NK cells aggravated MCD-induced fibrosis. **A** Livers of MCD-fed *UCP1*^flox/flox^*NCR1*^cre^ mice. **B** Serum AST and ALT, and liver TG, TC and FFAs of MCD-fed mice. **C** Fibrosis in livers stained by Masson and Sirius red. **D** Hepatic NK cells measured by flow cytometry. **E** Variations in NKG2D, CD69, IFN-γ of hepatic NK cells. **F** Macrophages in liver of MCD-fed mice detected by flow cytometry. **G** Diagram of endogenous NK cell depletion and infusion of exogenous NK cells. Serum levels of AST and ALT and liver TG, TC, and FFAs (**H**) of mice with infusion of exogenous NK cells. Fibrosis in livers stained by H&E, Masson, and Sirius red (**I**). (**J)** Variations of liver NK cells and macrophages. The experiments were carried out twice. Ns no significance; *P < 0.05; **P < 0.01; ***P < 0.001; ****P < 0.0001.

NCR1 is a surface marker of NK cells. Next, whether intrinsic deficiency of UCP1 in NK cells affects NHSH progression to fibrosis was determined in UCP1^flox/flox^-NCR1^cre^ (cKO) mice. As expected, MCD-fed cKO mice exhibited severe lipid accumulation in liver, hepatitis, and fibrosis (Fig. 2A–C). NK cells decreased in liver of cKO mice (Fig. 2D and Supplementary Fig. 2A), accompanied with reduced expression of NKG2D, CD69, and IFNγ, (Fig. 2E and Supplementary Fig. 2B). Concurrently, more M2 macrophages with elevated production of TNF-α and TGF-β1 expression were present in livers of cKO mice (Fig. 2F and Supplementary Fig. 2C, D). Then, WT mice were pre-depleted of NK cells, adoptively injected with NK^WT^ or NK^KO^ cells, and fed with MCD (Fig. 2G). Increased serum ALT and AST (Fig. 2H) and liver TG, TC, and FFAs (Fig. 2I) were observed in mice transfused with NK^KO^ cells. Histologic analysis showed increased droplets, blue fiber-like area (Masson), and red fiber-like region (Sirius red) in the fatty liver of NK^KO^ cell-transfused mice (Fig. 2J). Furthermore, less NKG2D^+^ NK cells and more CD206^+^ macrophages were identified in the fatty liver of mice transfused with NK^KO^ cells (Fig. 2K), as compared with infusion of NK^WT^ cells. These experiments confirmed that compromised activity of UCP1^−/−^ NK cells under high-lipid microenvironment facilitates hepatitis progression to liver fibrosis.

### UCP1^−/−^ NK cells of MCD-fed mice increase necroptosis with reduced mitophagy

**Fig. 3:**
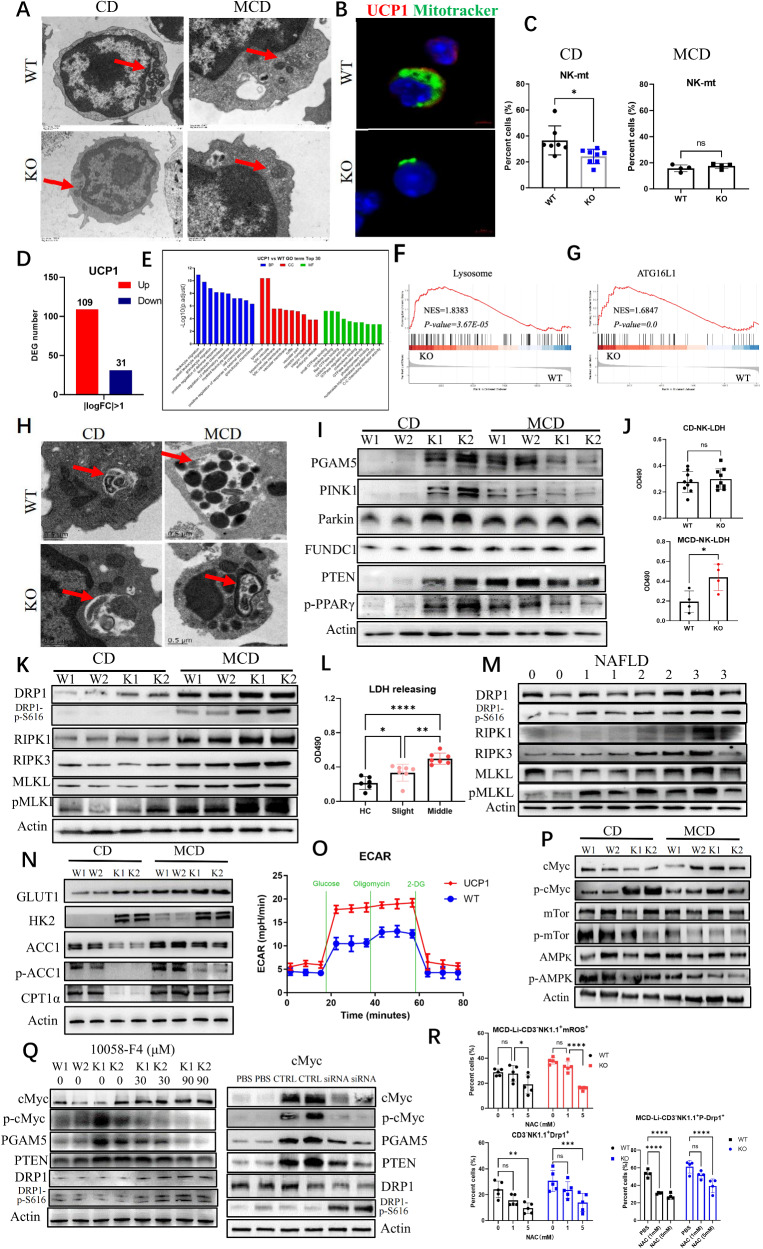
Variations of mitophagy and necroptosis in UCP1^−/−^ NK cells under high-lipid context. **A** Electronic microscope of mitochondria in NK cells. **B** Co-staining of UCP1 and mitotracker by confocal microscope. **C** Mitochondrial weight of NK cells upon CD or MCD feeding. **D** Differential expression genes (DEGs) between NK^WT^ and NK^KO^ cells physiologically. **E** GO analysis of DEGs. Changes of lysosome (**F**) and ATG16L1 (**G**) displayed by GSEA. **H** Autophagic vacuoles in NK cells upon CD or MCD feeding. Variations of mitophagy molecules (**I**), LDH releasing (**J**), and necroptosis molecules (**K**) in MCD-treated mice. LDH releasing (**L**) and necroptosis molecules (**M**) in NAFLD patients. **N** Rate-limiting enzymes involved in glycolysis and oxidative phosphorylation. **O** Glycolysis determined by the Seahorse analysis. **P** Detection of p-cMyc, p-mTOR, AMPK, and p-AMPK. **Q** Inhibiting p-cMyc on NK cell mitophagy and necroptosis. **R** Variation of p-DRP1^S616^ by ROS depletion (N-acetylcysteine, 5 mM) in UCP1^−/−^ NK cells of MCD-fed mice. NK cells were isolated from mouse spleen. The experiments were repeated at least twice. Ns no significance; *P < 0.05; **P < 0.01; ***P < 0.001; ****P < 0.0001.

Why the bioactivities of UCP1^−/−^ NK cells were compromised after mice were fed with MCD was explored. Given that UCP1 is located in the mitochondria, the NK cell morphology was observed by electron microscopy. Shrunken mitochondria were observed in physiologic UCP1^−/−^ NK cells and in NK^WT^ cells of mice fed with MCD (Fig. 3A). Confocal images showed that the UCP1-deficient NK cells had less mitochondrial staining (Fig. 3B). Lower mitochondrial mass of physiologic UCP1^−/−^ NK cells was also confirmed by flow cytometry (Fig. 3C). Although the difference in mitochondrial weight of NK cells between the two types of mice upon MCD feeding almost disappeared (Supplementary Fig. 3A), the mitochondrial masses of both NK cells dramatically decreased compared with those in the chow diet (CD) (Fig. 3C). There was not variation of mitochondrial biogenesis in UCP1^−/−^ NK cells, as shown in Supplementary Fig. 3B. Thus, UCP1 deficiency led to mitochondrial damages in NK cells upon MCD administration.

Next, the RNA transcriptomes of physiologic NK cells from WT and KO mice were compared. A total of 109 upregulated and 31 downregulated gene transcriptions were identified in the UCP1^−/−^ NK cells (Fig. 3D). Based on gene ontology analysis, the top five differentially expressed genes (DEGs) in cellular components were lysosome, lytic vacuole, lysosomal membrane, lytic vacuole membrane, and vacuolar membrane (Fig. 3E). Gene set enrichment analysis further demonstrated the increased lysosome and ATG16L1 signaling in UCP1^−/−^ NK cells (Fig. 3F, G), indicating that UCP1^−/−^ NK cells upregulated the autophagy activity. Obviously autophagic vacuoles were engulfed with shrunk mitochondrion in the physiologic UCP1^−/−^ NK cells, as detected by electronic microscopy (Fig. 3H). A notable detail that the NK^WT^ cells of mice fed with MCD also increased the mitochondrial autophagy (mitophagy). However, upon MCD administration, NK^KO^ cells cannot phagocytose the damaged mitochondrion efficiently because of some free damaged mitochondria in the cytoplasm (Fig. 3H).

Next, variations in the key molecules involved in mitophagy were examined. Upon CD feeding, the UCP1^−/−^ NK cells exhibited a substantially increase in the expression levels of PGAM5, PINK1, Parkin, and FUNDC1, accompanied with elevated PTEN and PPAR-γ (Fig. 3I). Upon MCD feeding, the normal NK cells also showed increased expression levels of mitophagy molecules, whereas the UCP1^−/−^ NK cells had substantially decreased expression levels of those molecules, indicating insufficient mitophagy in UCP1^−/−^ NK cells under the high-lipid microenvironment (Fig. 3I). Increased death of UCP1^−/−^NK cells, as determined by LDH release, was observed after MCD feeding (Fig. 3J). The necroptotic molecules (RIK1, RIK3, pMLKL, and pDRP1^S616^) in UCP1^−/−^ NK cells increased upon MCD administration (Fig. 3K) and increased mitochondrial fission mediated by pDRP1^S616^ (Supplementary Fig. 3E). Moreover, the LDH release rate and the necroptotic molecules of NK cells were elevated in patients with NAFLD^moderate^ (Fig. 3L, M). Thus, the UCP1-deficient NK cells are likely to enter the necroptotic state under the high-lipid condition, accompanied with reduced mitophagy.

Given that UCP1 is a mitochondrial molecule, UCP1 deficiency could affect cell metabolism. Hexokinase 2 (HK2), as a rate-limiting enzyme of glycolysis, was found to be substantially increased in physiologic UCP1^−/−^ NK cells, accompanied with decreased ACC1 (a rate-limiting enzyme of fatty acid synthesis) and CPT1α (a rate-limiting enzyme of fatty acid oxidation). However, upon MCD administration, the UCP1^−/−^ NK cells showed an increase in the expression levels of ACC1 and CPT1α, indicating increased metabolic activities in the mitochondria (Fig. 3N). Meanwhile, ECAR measurements revealed enhanced glycolysis in UCP1^−/−^ NK cells (Fig. 3O). NK cell glycolysis is regulated by c-Myc rather than mTOR [27]. The physiologic UCP1^−/−^ NK cells exhibited sharply enhanced c-Myc expression, and this expression was reduced by MCD (Fig. 3P). However, no increase in p-mTOR and p-AMPK was found in the UCP1^−/−^ NK cells upon CD or MCD feeding. Thus, the increase in c-Myc resulted from the adaptive metabolic reprogramming in NK cells due to the abnormal mitochondria induced by UCP1 deficiency. When c-Myc activation was inhibited by its inhibitor (10058-F4) or downregulated by its siRNA, the increased expression of PGAM5 and PTEN in UCP1^−/−^ NK cells restored, suggesting that the c-Myc activation was responsible for mitophagy (Fig. 3Q). However, both reagents increased DRP1^S616^ phosphorylation, indicating that the magnitude of c-Myc activation was a key factor for the transition from mitophagy to necroptosis of NK cells (Fig. 3Q). Upon MCD administration, the CD36 expression and mitochondrial ROS (mROS) in the liver UCP1^−/−^ NK cells increased (Supplementary Fig. 3C, D). Meanwhile, the pDRP1^S616^ level decreased when liver UCP1^−/−^ NK cells were depleted of cellular ROS by N-acetylcysteine (NAC), demonstrating that increased mROS was involved in necroptosis (Fig. 3R).

Collectively, UCP1 deficiency in NK cells induces mitophagy to maintain normal activity physiologically. However, when these UCP1^−/−^ NK cells are placed in a high-lipid condition, the increased fatty acid oxidation aggravates the irreversible damages of the mitochondria and then promotes cell necroptosis.

### UCP1 deficiency intensifies PA-induced necroptosis of UCP1^−/−^NK cells

**Fig. 4:**
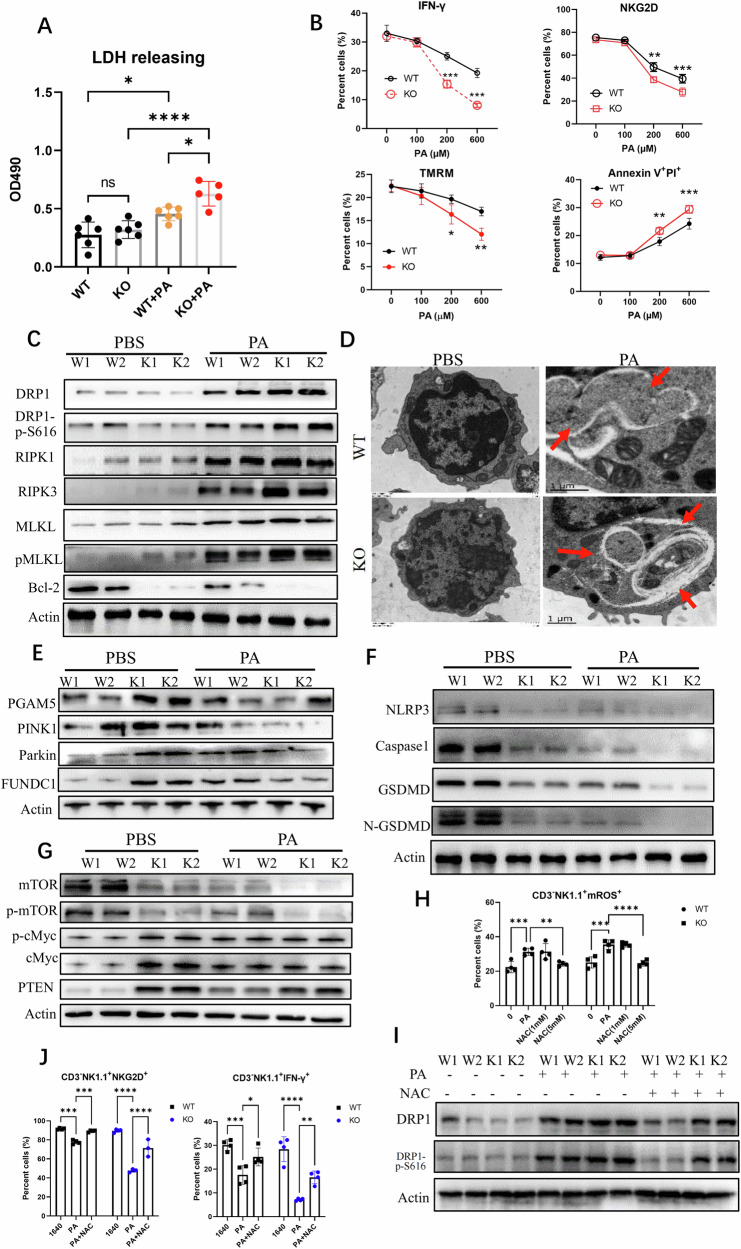
Mitophagy and necroptosis of UCP1^−/−^ NK cells treated with PA ex vivo. **A** LDH releasing of NK cells with the PA treatment. **B** NKG2D, IFN-γ, mitochondrial membrane potential (ψm) using tetramethylrhodamine methyl ester (TMRM), and apoptosis of NK cells treated with PA. **C** Necroptosis-associated molecules and Bcl-2 in PA-treated NK cells. **D** Mitochondrion and autophagic vacuoles in PA-treated NK cells. Variations of mitophagy (**E**), pyroptosis (**F**), and mTOR, cMyc, and PTEN (**G**) in NK cells before and after PA treatment. **H** ROS depletion in NK cells by NAC (5 mM). **I** Phosphorylated DRP1^S616^ of NK cells after the ROS depletion. **J** Detection of NK cell activity following NAC (5 mM) treatment. NK cells were isolated from mouse spleens. All experiments were repeated at least twice. Ns no significance; *P < 0.05; **P < 0.01; ***P < 0.001; ****P < 0.0001.

Whether NK cell death could be affected by PA was further checked ex vivo. The treatment of PA (200 μM) was able to induce the death of NK^WT^ cells, as indicated by the increased LDH release, and this dose of PA induced more efficient death of UCP1^−/−^ NK cells (Fig. 4A). As expected, PA decreased IFN-γproduction and mitochondrial membrane voltage and increased Annexin V/PI staining of NK^WT^ cells [28]. The same dose of PA induced deeper changes in UCP1^−/−^ NK cells. Although PA had no effects on the NKG2D of WT-NK cells, it reduced the NKG2D expression of UCP1^−/−^ NK cells (Fig. 4B and Supplementary Fig. 4A). In parallel, the necroptosis molecules (RIPK1, RIPK3, pMLKL, and pDRP1^S616^) in PA-treated UCP1^−/−^ NK cells increased more than those in NK^WT^ cells. The antiapoptotic molecule of the mitochondria, Bcl-2, almost disappeared in PA-treated UCP1^−/−^ NK cells (Fig. 4C). Meanwhile, no increase in apoptosis molecules, such as caspase-3, caspase-8, and caspase 9, was observed between the PA-treated NK^WT^ and NK^KO^ cells (Supplementary Fig. 4B). Meanwhile, ferroptosis-associated molecules, GPX4, ferritin, and Alox12, showed no differences between above NK cells (Supplementary Fig. 4C). Thus, UCP1 deficiency intensified NK cell necroptosis upon PA treatment ex vivo.

Whether the PA-induced UCP1^−/−^ NK cell necroptosis was involved with the reduced mitophagy was further analyzed. Free injured mitochondria were frequently present in the PA-treated UCP1^−/−^ NK cells (Fig. 4D). Simultaneously, although PA induced the expression of mitophagy molecules in NK^WT^ cells, a substantial reduction in the PINK1, Parkin, and FUNDC1 expression levels was shown in the UCP1^−/−^ NK cells. Despite the increase in PGAM5 expression of physiologic UCP1^−/−^ NK cells, this molecule was sharply reduced after PA treatment (Fig. 4E). More injured mitochondria due to insufficient mitophagy could generate ROS, which is possibly able to activate inflammasome for pyroptosis. However, no increase was found in the NLRP3, caspase-1, gasdermin D (GSDMD), and N-terminal of GSDMD in PA-treated UCP1^−/−^ NK cells (Fig. 4F).

Similar with liver NK cells from MCD-treated UCP1^−/−^ mice, decreased mTOR and p-mTOR were seen in UCP1^−/−^ NK cells, particularly in PA-treated UCP1^−/−^ NK cells. Although c-Myc/p-c-Myc was increased in normal UCP1^−/−^ NK cells as before, PA efficiently suppressed the c-Myc/p-c-Myc level of UCP1^−/−^ NK cells, confirming the role of c-Myc activation in regulating NK cell fate. PA can increase PTEN in NK^WT^ cells weakly, but it decreased the PTEN level of UCP1^−/−^ NK cells, reflecting more damages caused by PA in UCP1^−/−^ NK cells (Fig. 4G). Next, when the PA-treated UCP1^−/−^ NK cells were depleted of ROS by NAC (Fig. 4H), the pDRP1^S616^ level in NK^WT^ cells restored and reversion of NK cell activity was observed (Fig. 4I, J). Thus, UCP1^−/−^ NK cells are more prone to necroptosis upon PA treatment due to the decrease in c-Myc activation.

### Poly I:C can inhibit PA-induced necroptosis of UCP1^−/−^ NK cells independent of mitophagy

**Fig. 5:**
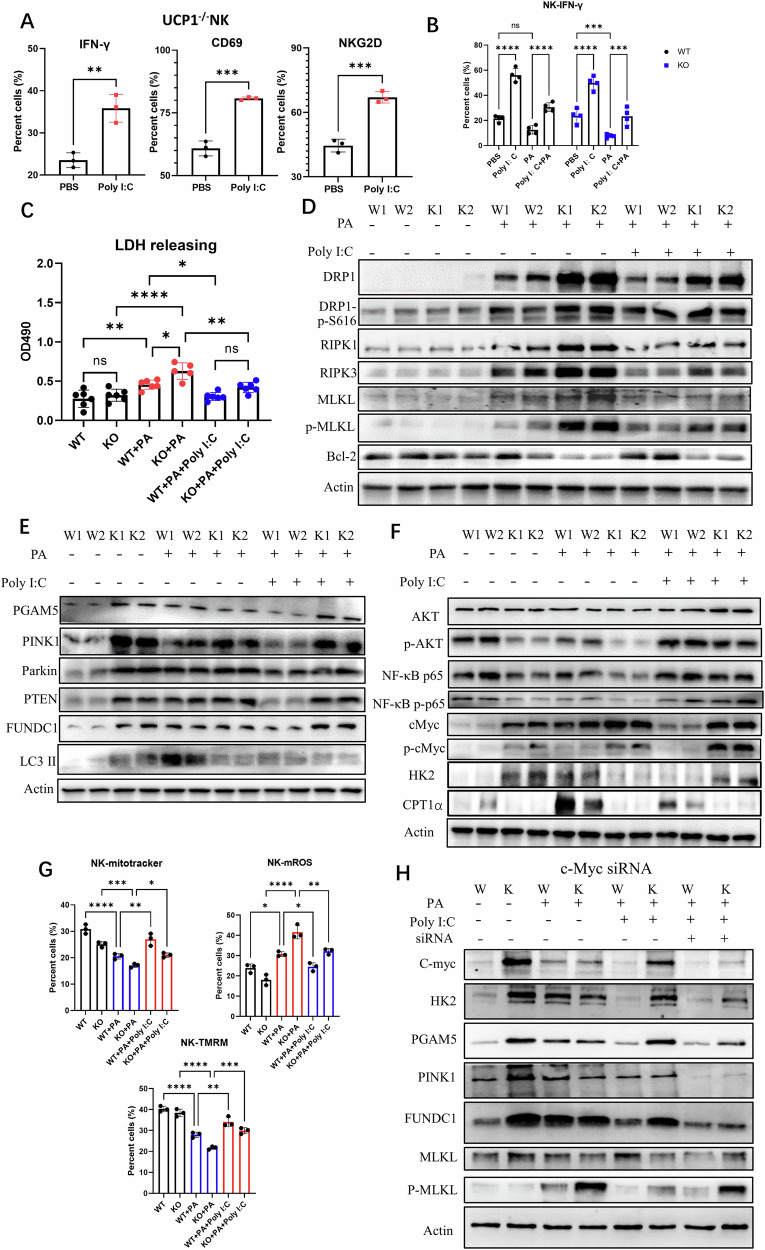
Necroptosis of PA-treated UCP1^−/−^ NK cells could be reversed by poly I:C. **A** IFN-γ, CD69, and NKG2D of UCP1^−/−^ NK cells stimulated with poly I:C. IFN-γ production (**B**), LDH releasing (**C**), necroptosis molecules and Bcl-2 (**D**), mitophagy molecules (**E**), and p-Akt, NF-κB p65, c-Myc, HK2, and CPT1α (**F**), mitotracker, mitoSOX and TMRM (**G**) in PA-treated NK^KO^ cells stimulated with Poly I:C. **H** Variations of molecules involved in mitophagy and necroptosis after splenic NK cells were treated with a cMyc siRNA. All experiments were repeated at least twice. Ns no significance; *P < 0.05; **P < 0.01; ***P < 0.001; ****P < 0.0001.

Given that poly I:C functions as a toll-like receptor 3 agonist, whether necroptosis of PA-treated UCP1^−/−^ NK cells could be reversed by poly I:C ex vivo were investigated. Clearly, poly I:C can efficiently promote the IFN-γ, CD69, and NKG2D expression of UCP1^−/−^ NK cells (Fig. 5A and Supplementary Fig. 5A). However, poly I:C stimulation cannot completely reverse the decrease in IFN-γ production induced by PA, either in NK^WT^ or NK^KO^ cells (Fig. 5B and Supplementary Fig. 5B). In terms of cell death, poly I:C was able to completely inhibit the LDH release of NK^WT^ or NK^KO^ cells induced by PA (Fig. 5C). Moreover, after poly I:C treatment was conducted, remarkable decreases in necroptotic molecules (pDRP1^S616^, RIPK1, RIPK3, and pMLKL) [29] with no changes in Bcl-2 were observed in UCP1^−/−^ NK cells (Fig. 5D), demonstrating that poly I:C exerted a protective role against cell death.

Next, the mitophagy molecules of poly I:C-treated NK cells under high-lipid microenvironment were examined. As shown in Fig. 5E, poly I:C/PA stimulation did not obviously increase the levels of PGAM5, PINK1, Parkin, PTEN, FUNDC1, and LC3 II in UCP1^−/−^ NK cells [30] compared with PA treatment alone. A notable detail that PA promoted the expression levels of these mitophagy molecules in NK^WT^ cells, and the co-stimulation of poly I:C conversely downregulated their expression levels (Fig. 5E). Poly I:C was still able to stimulate the AKT, NF-κB p65, and cMyc phosphorylation of PA-treated NK^KO^ cells (Fig. 5F). Given that cMyc activation can promote NK cell glycolysis, increased HK2 and alleviated Mitochondrial damage was observed in PA-treated UCP1^−/−^ NK cells after the co-stimulation of poly I: C. However, these NK cells retained a low level of CPT1α (Fig. 5F, G), and when c-Myc was inhibited, UCP1−/− NK cell glycolysis decreased, leading to necroptosis (Fig. 5H), confirming the key role of c-Myc activation on regulating NK^KO^ cell death. Also, when an autophagy-stimulating reagent, mitochonic acid-5 (MA5) or valproic acid (VPA), was added to the PA-treated UCP1^−/−^ NK cells [31, 32], no obvious restoration in NK cell activities was found, as suggested by cell death, CD69, NKG2D, IFN-γ, and granzyme B (Supplementary Fig. 5D). Thus, poly I:C enhanced the glycolysis of NK cells to avoid mitochondrial damages in UCP1^−/−^NK cells rather than stimulating mitophagy.

### Rescued expression of UCP1 in UCP1^−/−^ NK cells inhibits liver fibrosis

**Fig. 6:**
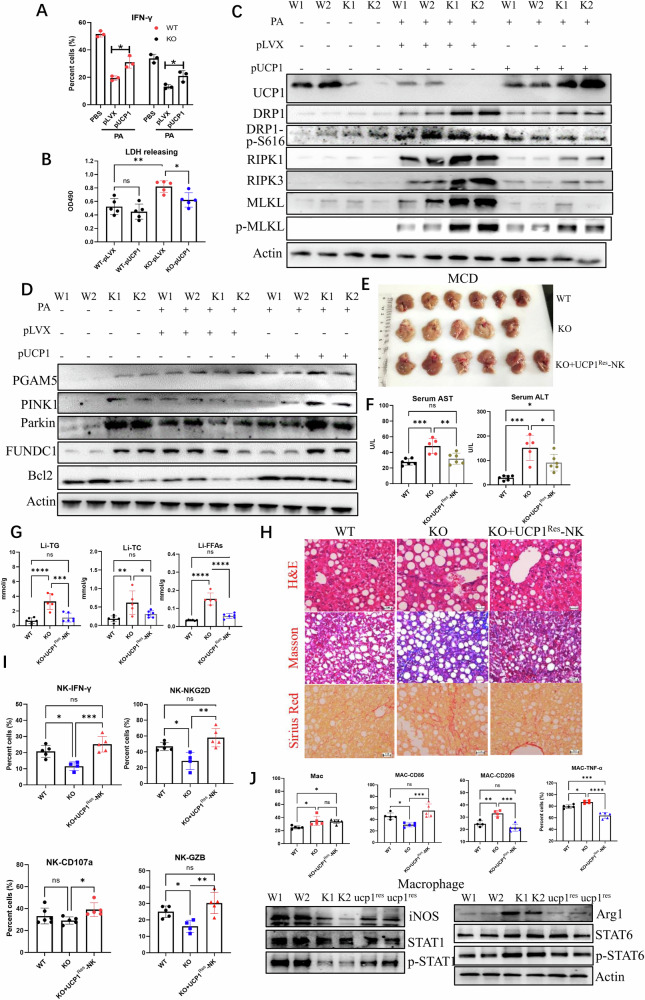
Rescue of UCP1 restored NK cell activities. IFN-γ production (**A**) and LDH releasing (**B**) of splenic NK cells after being transfected with UCP1. Necroptosis (**C**) and mitophagy (**D**) molecules in splenic NK cells with the rescue of UCP1. Livers (**E**), serum AST and ALT levels (**F**), liver TG, TC, and FFAs (**G**), and fibrosis stained by H&E, Masson, and Sirius red (**H**) in livers of MCD-fed mice with the transfusion of UCP1-rescued NK cells. Activities of liver NK cells (**I**) and macrophages (**J**). All experiments were carried out at least twice. Ns no significance; *P < 0.05; **P < 0.01; ***P < 0.001; ****P < 0.0001.

The capability of IFN-γ production of PA-treated UCP1^−/−^ NK cells can be reversed when the NK^KO^ cells were rescued to express UCP1 (Fig. 6A and Supplementary Fig. 6A). These UCP1-rescued NK^KO^ cells also decreased the LDH release induced by PA treatment, reflecting the decreased death of NK cells (Fig. 6B). Also, after being rescued to express UCP1, NK^KO^ cells almost lost PA-induced p-DRP1, RIPK1, RIPK3, and p-MLKL (Fig. 6C). Meanwhile, the PA-induced decrease in the expression levels of PGAM5, PINK1, Parkin, FUNDC1, and Bcl-2 in NK^KO^ cells was restored when UCP1 was rescued (Fig. 6D). Although loss of UCP1 limited NK activity under PA treatment, no changes in activities was observed when UCP1 was over-expressed in NK^WT^ cells (Supplementary Fig. 6B).

**Fig. 7:**
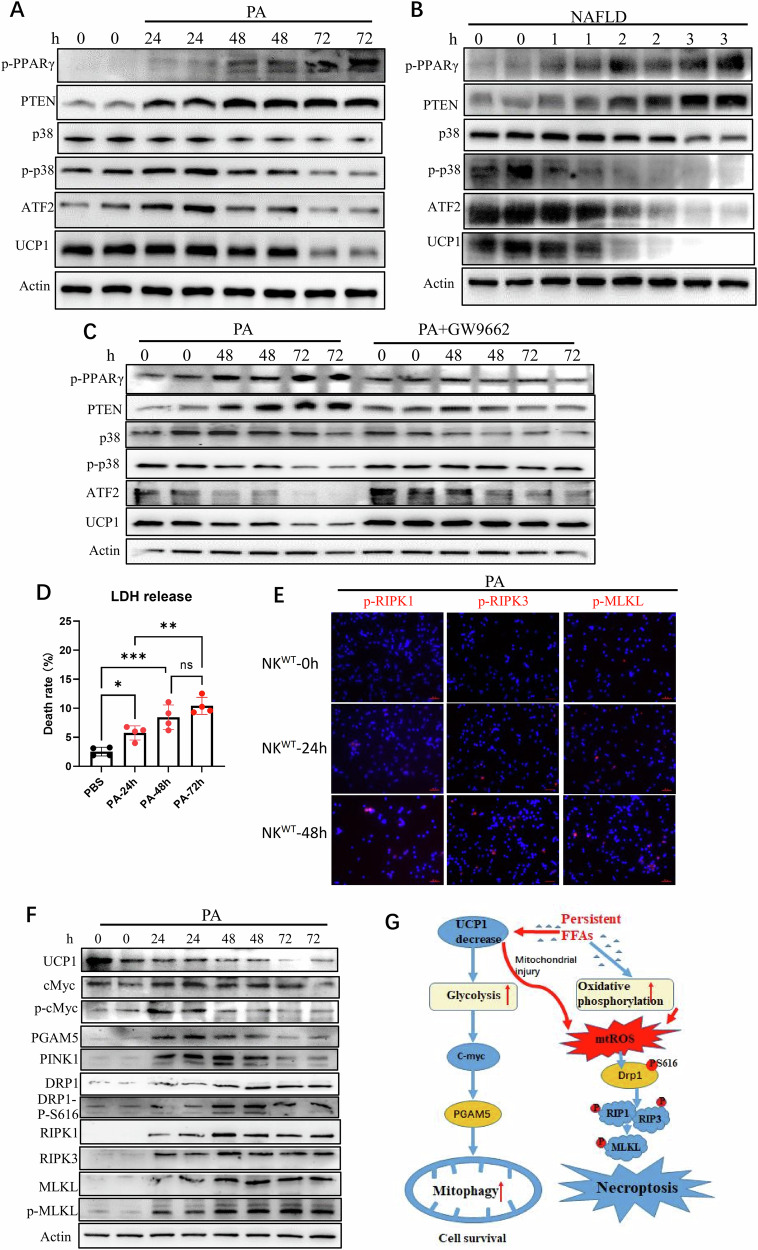
Decrease in UCP1 by sustained high PA promoted NK cell necroptosis in synergism of PA. Decreased UCP1 in murine NK cells by PA (600 μM) treatment for 24-, 48-, or 72-h (**A**) and in NALFD patients-derived NK cells (**B**) in association with PPARγ, PTEN, p38, and ATF2. **C** UCP1 in PA-treated NK cells before and after in coculture with a PPARγ inhibitor (GW9662). **D** LDH releasing of NK cells after PA treatment. **E** Immunofluorescence of RIPK1, RIPK3, and p-MLKL of NK cells with the PA treatment for 48 h. **F** Dynamic expressions of UCP1, mitophagy molecules, and necroptosis molecules in NK cells by the PA (600 μM) treatment for 72 h. **G** The working model of sustained high PA to downregulate NK cell bioactivity. *P < 0.05; **P < 0.01; ***P < 0.001; ****P < 0.0001.

Then, whether the adoptive transfer of UCP1-rescued UCP1^−/−^NK cells could suppress the progression of NAFLD in KO mice fed with MCD was explored. As shown in Fig. 7E, when the UCP1^res^ NK cells were intravenously transferred into KO mice, the lipid accumulation in the liver decreased. The serum levels of AST and ALT also decreased in these mice (Fig. 6F), accompanied with similar changes in the liver TCs, TGs, and FFAs (Fig. 6G). Histological analysis displayed a decrease in lipid droplets and obvious amelioration of fibrosis in the liver of mice transferred with UCP1^res^ NK cells (Fig. 6H). The liver NK cells from mice with transfusion exhibited an increase in NKG2D, IFN-γ, GZB, and degranulation (CD107a; Fig. 6I and Supplementary Fig. 6C). Simultaneously, Increased CD86^+^ macrophages and decreased CD206^+^ or TNF-α^+^ macrophages were present in the liver of UCP1^res^-NK cell-transferred mice. These macrophages had high production of p-STAT1 and iNOS, whereas low expression of ARG1 and p-STAT6 (Fig. 6J and Supplementary Fig. 6D). Collectively, these results demonstrated that rescued expression of UCP1 in UCP1^−/−^ NK cells can restore NK cell bioactivities.

### Sustained high PA decreases UCP1 of NK cells and promotes NK cell necroptosis

UCP1 transcription is stimulated by p38 MAP kinase and its downstream ATF2. The cis-acting element of ATF2 is in the promoter region of UCP1 [33]. When normal NK cells were treated with high-dose PA (600 μM) within 24 h, no remarkable changes were observed in UCP1 expression. However, when the PA treatment was sustained in 48–72 h, a substantial decrease in UCP1 was seen in NK cells. A high level of PA was previously demonstrated by this group to efficiently induce PPAR-γ expression in NK cells [28]. With more than 48 h of PA stimulation, the NK cells showed increased expression of p-PPAR-γ. At this time, high PTEN with decreased p-p38 and ATF2 was seen in the NK cells (Fig. 7A). Similarly, increased p-PPAR-γ/PTEN with decreased p38/ATF2/UCP1 was demonstrated in the peripheral NK cells of patients with advanced NAFLD (Fig. 7B). When these PA-stimulated NK cells were co-treated with an inhibitor of PPAR-γ (GW9662), PTEN expression was impaired, and the levels of p38, ATF2, and UCP1 were maintained (Fig. 7C). Thus, sustained high PA inhibited UCP1 expression in NK cells through the PPAR-γ/PTEN/p38/ATF2 axis.

Considering that NK cell bioactivity is downregulated by the PA-induced PPAR-γ/PTEN/mTOR axis [28], the decreased UCP1 expression in NK cells by sustained PA can synergize with PA itself to promote NK cell necroptosis in an indirect manner. As expected, with 48–72-h stimulation of PA, necroptosis of NK cells was clearly observed, as demonstrated by increased LDH release (Fig. 7D) and expression of necroptotic molecules (Fig. 7E). Meanwhile, these PA-treated NK cells demonstrated an increase in the expression levels of p-cMyc, PGAM5, and PINK1 after 24 h and a decrease from 48 h (Fig. 7F). Thus, sustained PA treatment exerted profound inhibitions on NK cell bioactivity.

## Discussion

Downregulated NK cell bioactivity with decreased UCP1 was identified in patients with advanced NAFLD. Although no variated number and function of NK cell were seen in physiologic UCP1^−/−^ mice, compromised NK cell bioactivity was involved in the acerbation of NASH and liver fibrosis in MCD-fed UCP1^−/−^ mice. Progression of liver fibrosis either in MCD-fed UCP1^flox/flox^-NCR1^cre^ mice or in mice transfused with UCP1^−/−^ NK cells confirmed that a key role of cell-intrinsic deficiency of UCP1 on NK cell bioactivity. Mitochondrial injuries and increased mitophagy were present in MCD-fed NK^WT^ cells, PA-treated NK^WT^ cells, or physiologic NK^KO^ cells. The enhancement of mitophagy in those NK cells was attributed to metabolic reprogramming of glycolysis and increased c-Myc and PGAM5 expression. However, when the UCP1^−/−^ NK cells were placed in a high-lipid environment, necroptosis was induced by DRP1^S616^ activation accompanied with reduced mitophagy. Finally, persistent high PA can directly downregulate UCP1 expression via the PPAR-γ/PTEN/p38/ATF2 signaling pathway. Thus, persistent high lipid not only decreases UCP1 expression but also synergizes with UCP1 decrease to promote NK cell necroptosis and then facilitate NASH progression to fibrosis.

NK cell bioactivity can be downregulated under high-lipid condition via the upregulation of CD36-mediated intake of fatty acids and thus promote the PPAR-γ-mediated downregulation of Akt/mTOR [28]. In the present study, the NK cells were dually suppressed by sustained high-lipid-induced PPAR-γ activation. One effect is the inhibition of Akt/mTOR, which is a key regulator for NK cell activation; another effect is the decrease in p38/ATF2/UCP1, which is involved in NK cell death. The −3826 A/G polymorphism in the promoter of UCP1 is associated with obesity [34], type 2 diabetes [35], and hypertension [36]. In particular, the LDL-to-HDL CHO ratio was increased in the order of AA < AG < GG types in Korean subjects with obesity [37]. A single-nucleotide polymorphism of UCP2 (rs659366) is strongly associated with severe liver fibrosis [38]. Therefore, the −3826 A/G polymorphism of UCP1 could affect the biological function of NK cells and the severity of liver fibrosis under high-lipid condition.

A dynamic change in NK cell function is present in the progression of NAFLD [8, 9]. Enhanced NK cell activity aggravates liver inflammation (NASH) in the early phase, but liver fibrosis is an irreversible pathological injury with decreased NK cell activity. In MCD-induced NAFLD of UCP1^−/−^ mice, only the UCP1^−/−^ NK cells decrease in the liver (Fig. 1H–J), with no obvious changes in CD4^+^ or CD8^+^ T cells (Supplementary Fig. 1J), suggesting that NK cells were vulnerable under high-lipid stimulation. The NK cells cannot only kill activated hepatic stellate cells but also promote M1-polarized macrophage via IFN-γ production [10]. Thus, maintaining NK cell bioactivity could suppress the progression of nonalcoholic liver fibrosis. Meanwhile, considering the profound inhibitory effects of PPAR-γ activation on NK cells, the side effects of PPAR-γ agonists used in tumor therapy [39, 40] should be considered.

In conclusion, an important ability of NK cells to inhibit the progression of NASH toward fibrosis was emphasized here. NK cell bioactivity can be dually inhibited by persistent high lipid. Considering that the serum FFAs of a healthy human body are between 300 and 900 μM with PA of ~100 μM, balanced diet and appropriate physical exercises are encouraged to maintain normal lipid levels and avoid compromised NK cell function.

### Supplementary information

Supplementray table

Supplementray figures

Western Blot-original

## Data Availability

All data supporting this study are present in the paper and Supplementary Materials.
