# Supplementary material for: Decrease in UCP1 by sustained high lipid promotes NK cell necroptosis to exacerbate nonalcoholic liver fibrosis
Source: Cell Death Dis. 2024 Jul 20;15(7):518. doi: 10.1038/s41419-024-06910-4 (PMC11271447; doi:10.1038/s41419-024-06910-4)
Supplement: Supplementary file 1 — Supplementray table [file 41419_2024_6910_MOESM1_ESM.docx]

**Supplementary Table 1. Clinical characteristics of NAFLD patients and healthy controls**

| **Variables** | **Healthy (n=15)** | **Slight (n=16)** | **Middle（n=16）** |
| --- | --- | --- | --- |
| Age (years) | 51.53±4.72 | 52±3.354 | 42.69±3.151 |
| Male (%) | 66.67% | 62.5% | 93.75% |
| ALT (U/L) | 23.53±2.096 | 57.37±6.55 | 92.98±8.260 |
| AST (U/L) | 19.81±1.092 | 39.27±3.048 | 52.61±4.711 |
| GGT (U/L) | 25.72±2.950 | 55.39±6.444 | 67.9±6.752 |
| Bilirubin (umol/l) | 13.15±0.972 | 14.91±1.49 | 20.11±2.441 |
| Albumin (g/l) | 43.97±0.598 | 44.65±0.467 | 47.05±0.391 |
| Globulin（g/l） | 29.19±1.072 | 32.48±1.254 | 31.67±0.887 |
| Glucose（mmol/l） | 5.434±0.189 | 7.593±0.981 | 6.095±0.321 |
| Total cholesterol（mmol/l） | 4.741±0.249 | 5.136±0.190 | 4.92±0.180 |
| Triglyceride（mmol/l） | 1.564±0.157 | 2.079±0.223 | 2.975±0.460 |
| HDL-c（mmol/l） | 1.229±0.096 | 1.191±0.065 | 0.987±0.057 |
| LDL-c（mmol/l） | 2.765±0.171 | 2.945±0.185 | 2.827±0.193 |
| Platelet count (×10^9^/l)  (PLT) | 206.3±15.73 | 227.8±15.14 | 211.5±14.48 |
| Leukocyte’s count (×10^9^/l) | 6.041±0.397 | 6.175±0.507 | 6.344±0.426 |
| Lymphocyte count (×109/l) | 2.071±0.155 | 2.113±0.217 | 2.269±0.167 |

ALT, alanine aminotransferase; AST, aspartate aminotransferase; GGT, gamma-glutamyl transferase; HDL-c, high density lipoprotein cholesterol; LDL-c, low density lipoprotein cholesterol.
