# Supplementary material for: Decrease in UCP1 by sustained high lipid promotes NK cell necroptosis to exacerbate nonalcoholic liver fibrosis
Source: Cell Death Dis. 2024 Jul 20;15(7):518. doi: 10.1038/s41419-024-06910-4 (PMC11271447; doi:10.1038/s41419-024-06910-4)
Supplement: Supplementary file 2 — Supplementray figures [file 41419_2024_6910_MOESM2_ESM.pdf]

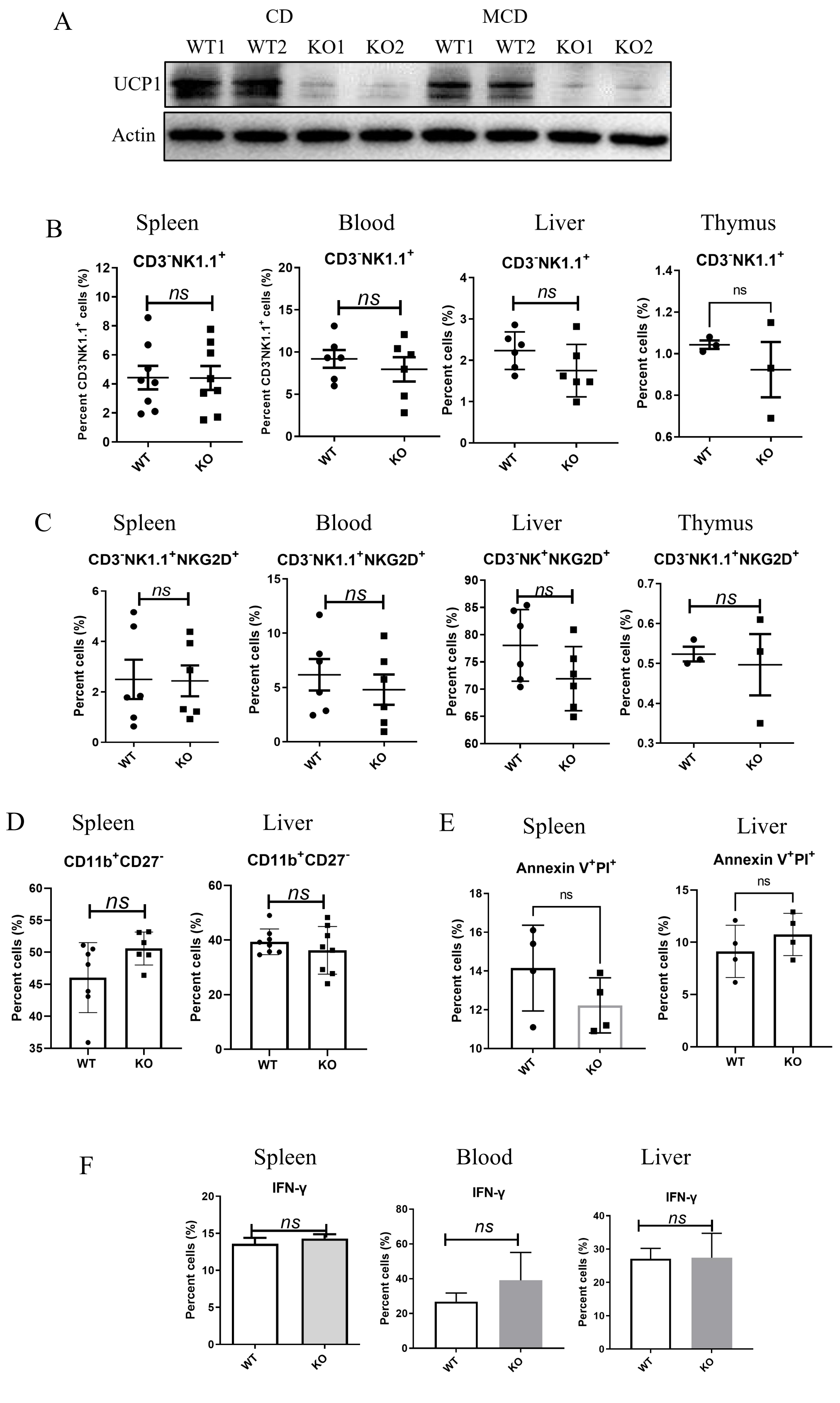

A
CD
MCD
WT1 WT2 KO1 KO2 WT1 WT2 KO1 KO2
UCP1
Actin
B
Spleen
Blood
Liver
Thymus
CD3⁻NK1.1⁺
Percent CD3⁻NK1.1⁺ cells (%)
Percent cells (%)
ns
WT
KO
C
Spleen
Blood
Liver
Thymus
CD3⁻NK1.1⁺NKG2D⁺
CD3⁻NK⁺NKG2D⁺
Percent cells (%)
ns
WT
KO
D
Spleen
Liver
CD11b⁺CD27⁻
Percent cells (%)
ns
WT
KO
E
Spleen
Liver
Annexin V⁺PI⁺
Percent cells (%)
ns
WT
KO
F
Spleen
Blood
Liver
IFN-γ
Percent cells (%)
ns
WT
KO

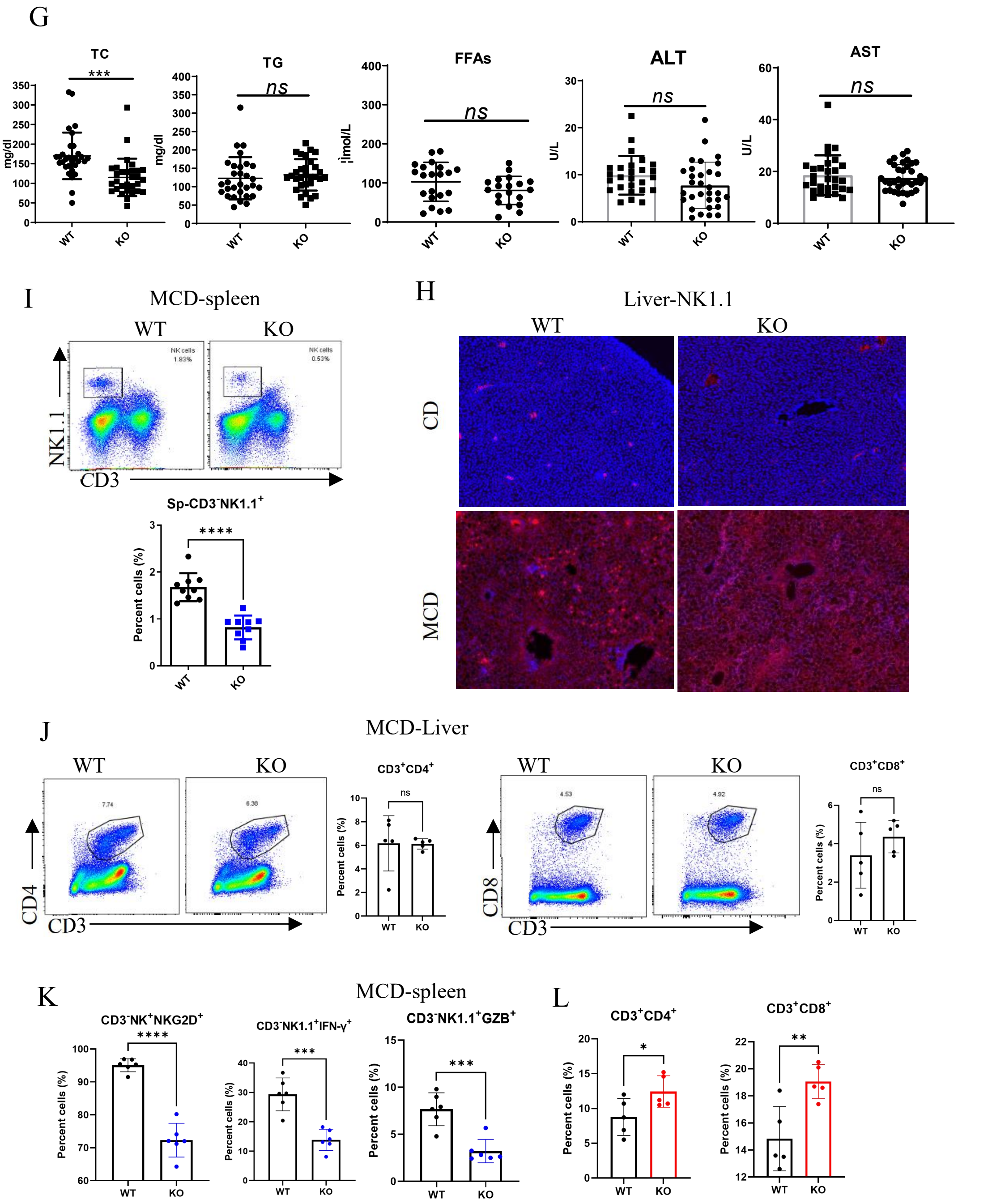

Supplementary Figure 1. Downregulation of NK cells in MCD-fed UCP1$^{-/-}$mice. (A) Confirmation of UCP1-deficiency in NK cells and decreased UCP1 of NK cells in WT mice fed with MCD. (B-F) Phenotype, differentiation, and activity of UCP1$^{-/-}$NK cells. (G) Serum cholesterol, TG, FFAs, ALT and AST in physiologic WT and KO mice. NK cells in spleens of MCD-fed mice checked by histological immune fluorescence (H) or flow cytometry (I). (J) Liver CD4$^+$ T and CD8$^+$ T cells in mice fed with MCD. Variations of NK cell (K) and CD4$^+$ T and CD8$^+$ T cells in spleens of mice fed with MCD. The experiments were repeated at least twice. Ns, no significance; * *P*<0.05; ** *P*<0.01; *** *P*<0.001.

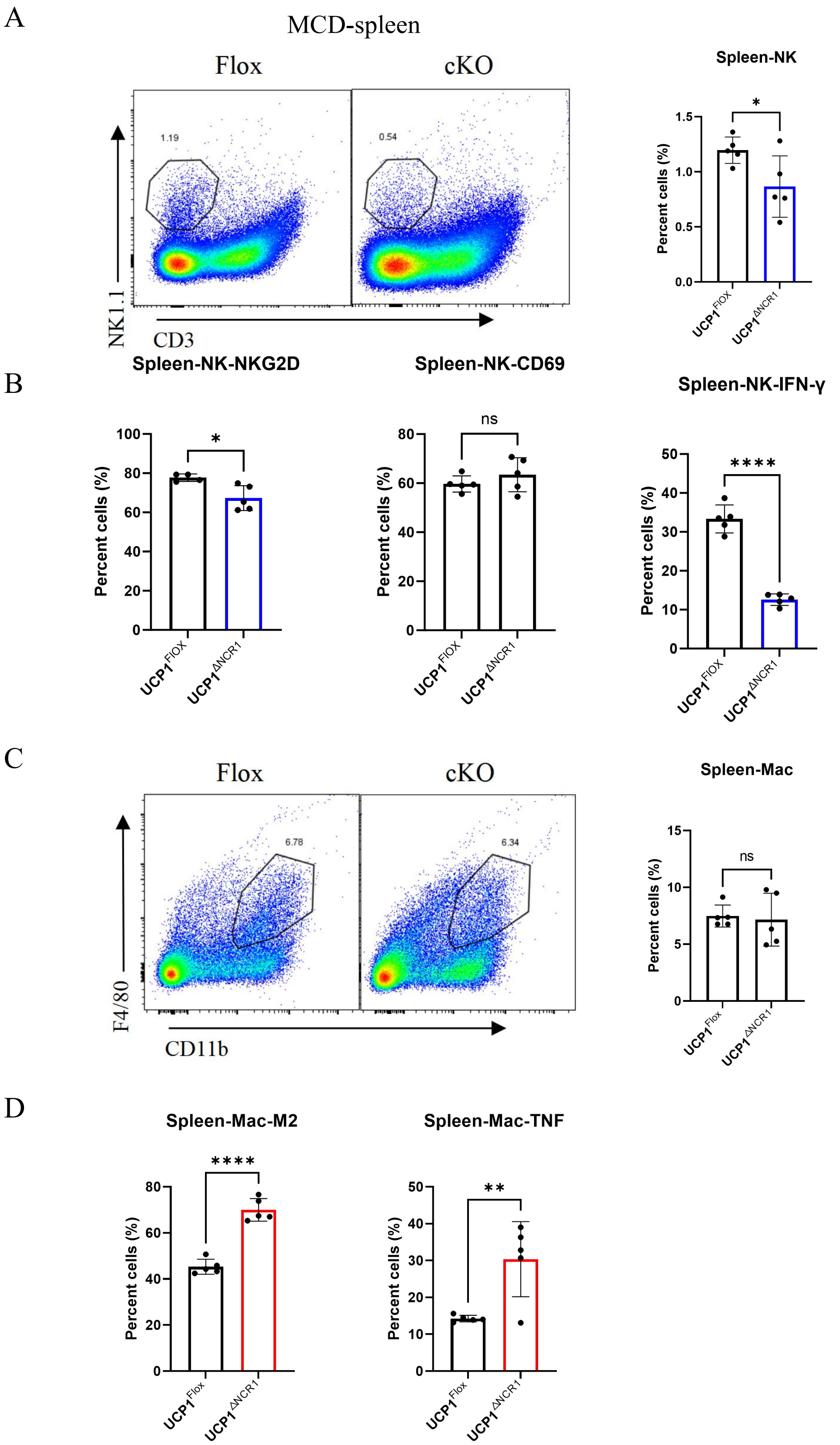

Supplementary Figure 2. NK cells in spleens of MCD-fed mice checked by flow cytometry (A). Alteration in NK cell activity (B). frequency of macrophages in spleens of MCD-fed mice (C) and activity (D).

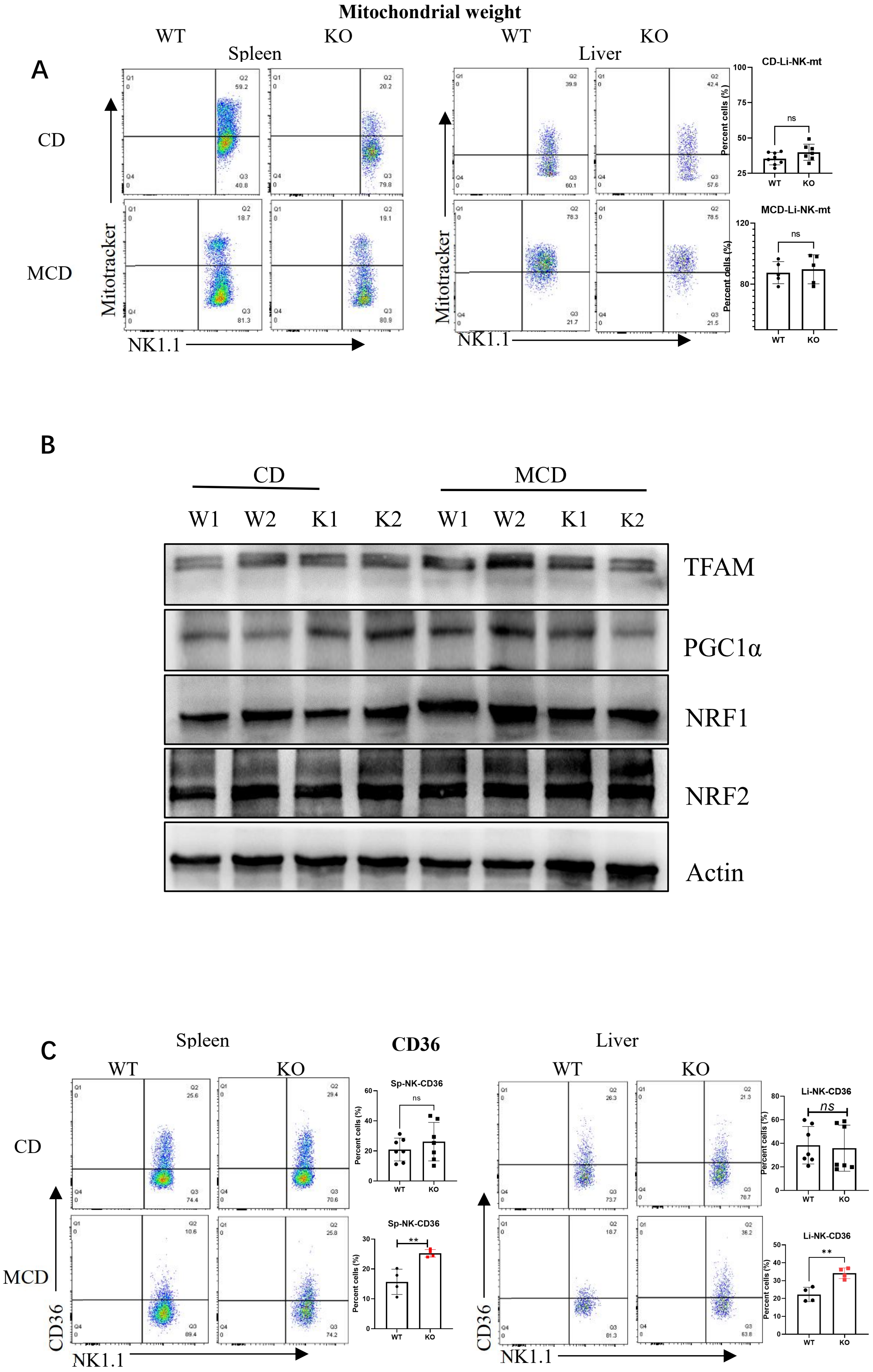

A
Mitochondrial weight
Spleen
Liver
WT
KO
CD
MCD
Mitotracker
NK1.1
CD-Li-NK-mt
MCD-Li-NK-mt
Percent cells (%)
ns
B
CD
MCD
W1
W2
K1
K2
TFAM
PGC1α
NRF1
NRF2
Actin
C
CD36
Spleen
Liver
Sp-NK-CD36
Li-NK-CD36
**

D

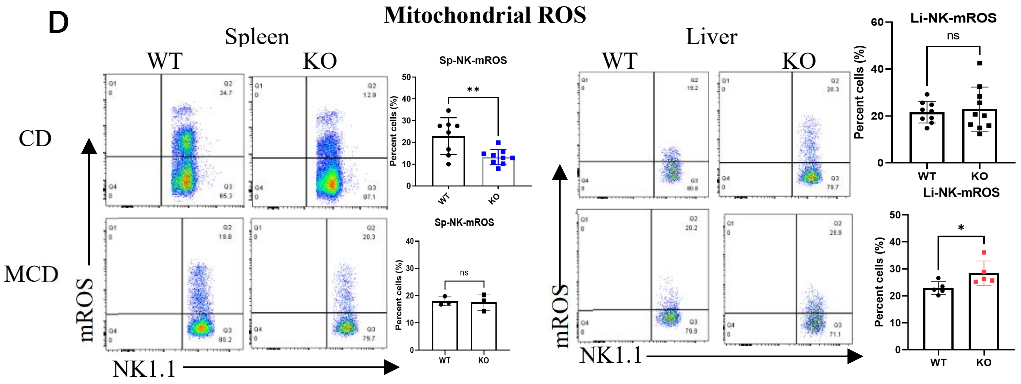

E

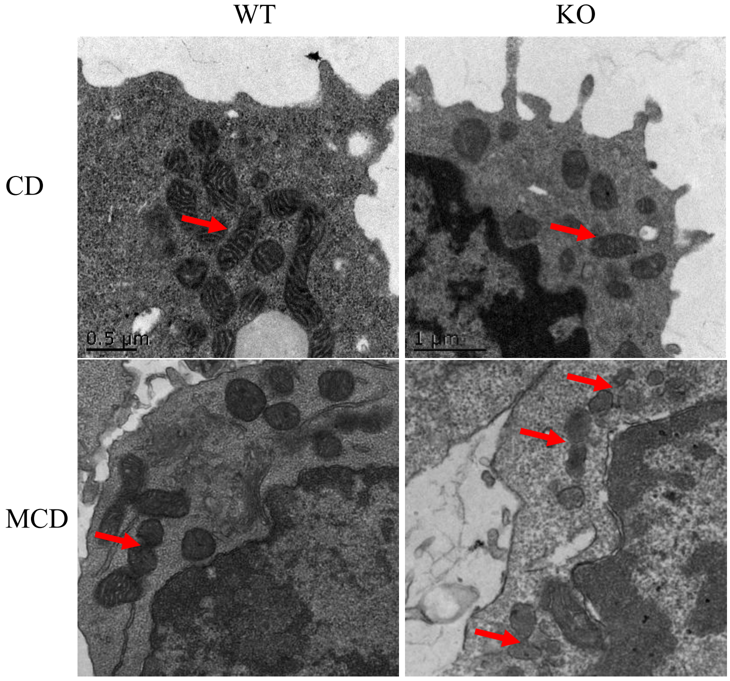

Supplementary Figure 3. Variations of mitochondrial weight (A), Mitochondrial biogenesis (B), CD36 (C), mROS (D) and Mitochondrial fission (E) of NK cells in mice upon CD or MCD feeding.

A

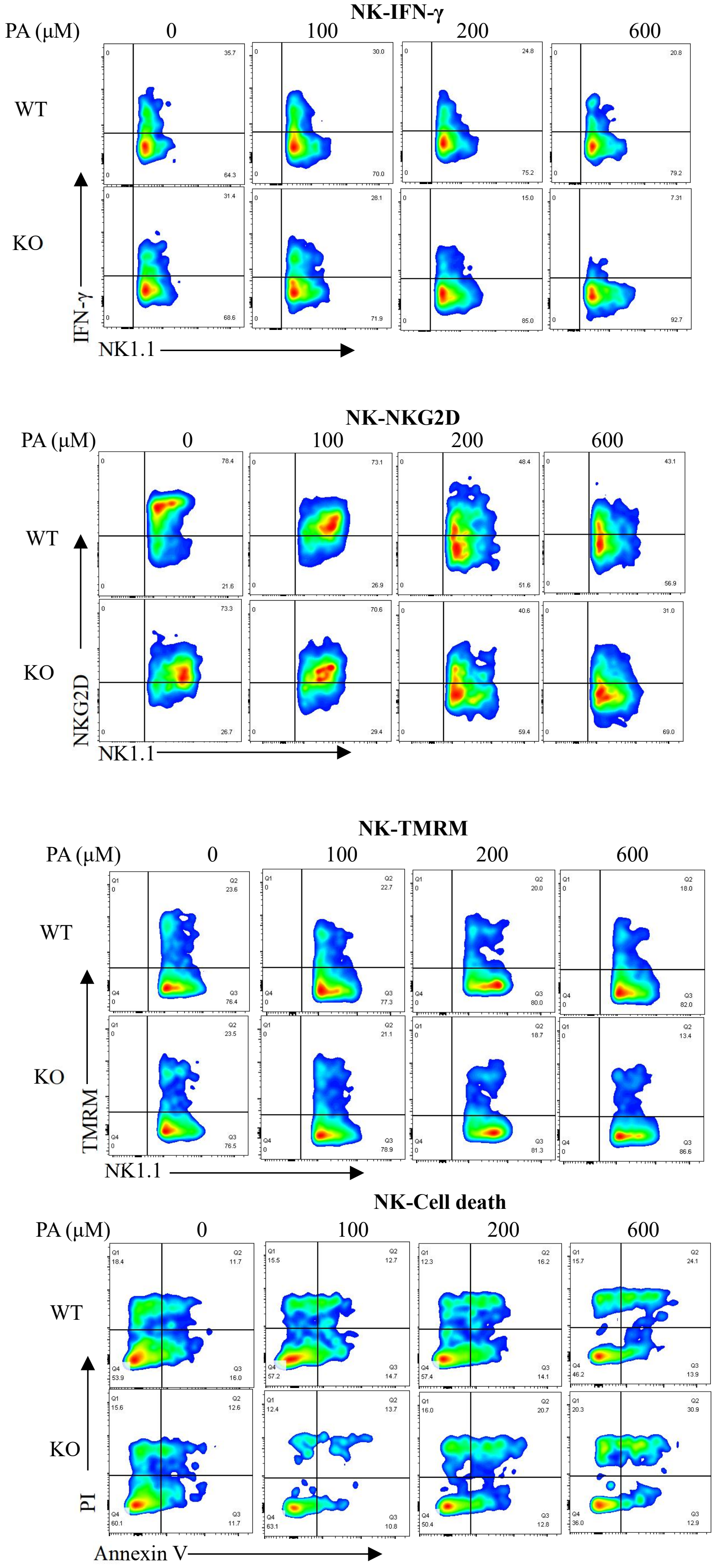

NK-IFN-γ
PA (μM)
0
100
200
600
WT
KO
IFN-γ
NK1.1
NK-NKG2D
PA (μM)
0
100
200
600
WT
KO
NKG2D
NK1.1
NK-TMRM
PA (μM)
0
100
200
600
WT
KO
TMRM
NK1.1
NK-Cell death
PA (μM)
0
100
200
600
WT
KO
PI
Annexin V

B

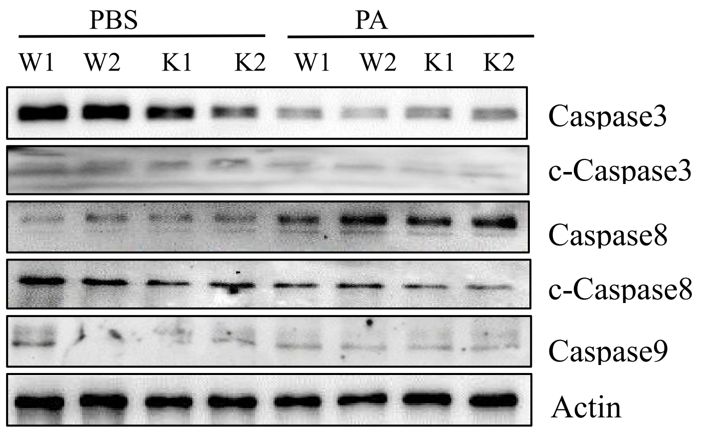

C

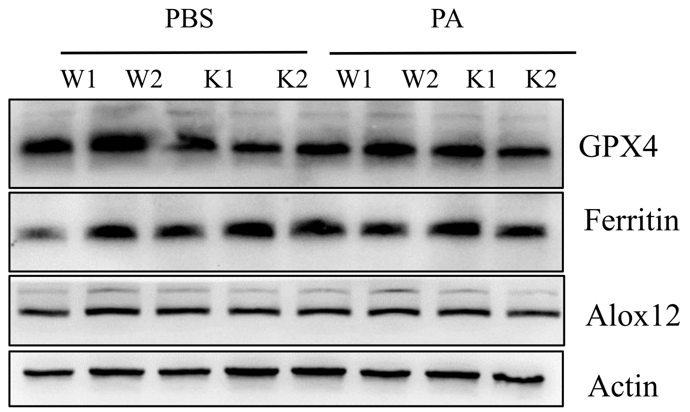

Supplementary Figure 4. Variations of NK cell activities by the PA treatment. (A) IFN-γ, NKG2D, TMRM, apoptosis of NK cells detected by flow cytometry. (B) Caspase 3, 8, 9 of NK cells treated with PA analyzed by western blot. (C) GPX4, Ferritin, Alox12 of NK cells treated with PA analyzed by western blot.

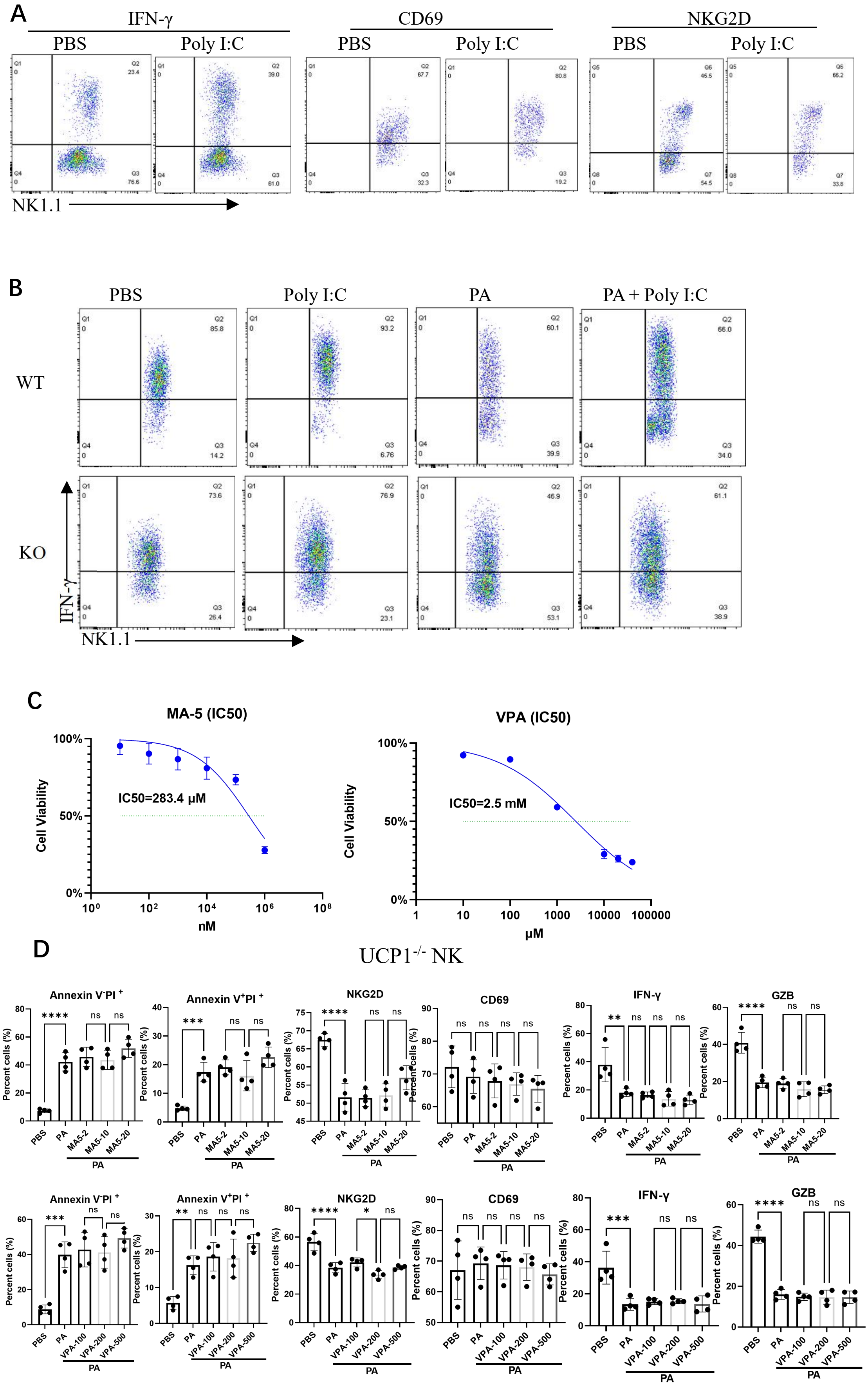
A
IFN-γ
CD69
NKG2D
PBS
Poly I:C
NK1.1
B
PBS
Poly I:C
PA
PA + Poly I:C
WT
KO
IFN-γ
NK1.1
C
MA-5 (IC50)
IC50=283.4 μM
Cell Viability
nM
VPA (IC50)
IC50=2.5 mM
μM
D
UCP1-/- NK
Annexin V-PI +
Annexin V+PI +
NKG2D
CD69
IFN-γ
GZB
Percent cells (%)
PBS
PA
MA5-2
MA5-10
MA5-20
VPA-100
VPA-200
VPA-500

Supplementary Figure 5. Representative results of poly I:C on NK cell activation. (A) Representative stimulation of UCP1$^{-/-}$ NK cells by poly I:C. (B) Effects of poly I: C on PA-treated NK cells. (C) IC50 of MA-5 or VPA on NK cells. (D) Effects of MA5 or VPA on NK cell activities.

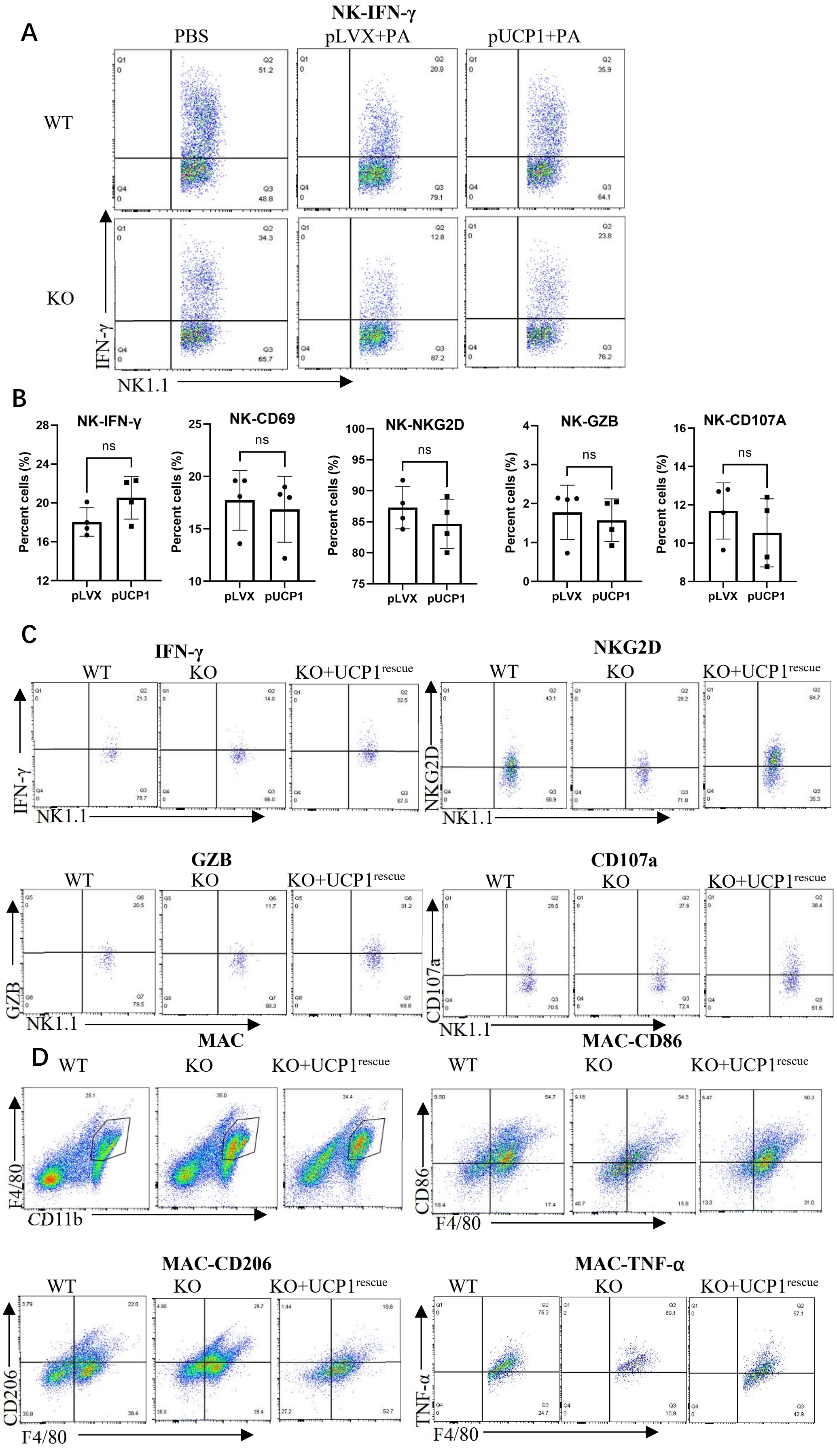

A
NK-IFN-γ
PBS
pLVX+PA
pUCP1+PA
WT
KO
IFN-γ
NK1.1
B
NK-IFN-γ
NK-CD69
NK-NKG2D
NK-GZB
NK-CD107A
Percent cells (%)
ns
pLVX
pUCP1
C
IFN-γ
NKG2D
GZB
CD107a
WT
KO
KO+UCP1rescue
NK1.1
D
MAC
MAC-CD86
MAC-CD206
MAC-TNF-α
F4/80
CD11b
CD86
CD206
TNF-α

Supplementary Figure 6. Rescue expression of UCP1 on NK cell activation. (A) IFN-γ production of PA-treated UCP1$^{-/-}$NK cells after the UCP1 transfection. (B) Variations of NK^{WT} cell activities by the UCP1 overexpression. Representative results of liver NK cell (C) and macrophage (D) activities in MCD-fed mice infused with UCP1-rescued NK cells.
