# Supplementary material for: Decrease in UCP1 by sustained high lipid promotes NK cell necroptosis to exacerbate nonalcoholic liver fibrosis
Source: Cell Death Dis. 2024 Jul 20;15(7):518. doi: 10.1038/s41419-024-06910-4 (PMC11271447; doi:10.1038/s41419-024-06910-4)
Supplement: Supplementary file 3 — Western Blot-original [file 41419_2024_6910_MOESM3_ESM.docx]

Figure 1D-UCP1


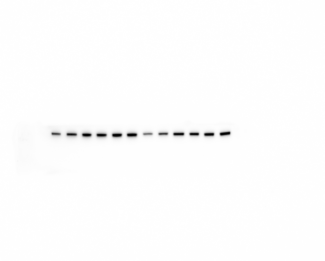


Figure 1B-UCP1

Figure 1B-Actin

Figure 1D-Actin








Figure 1E-Actin

Figure 1E-UCP1


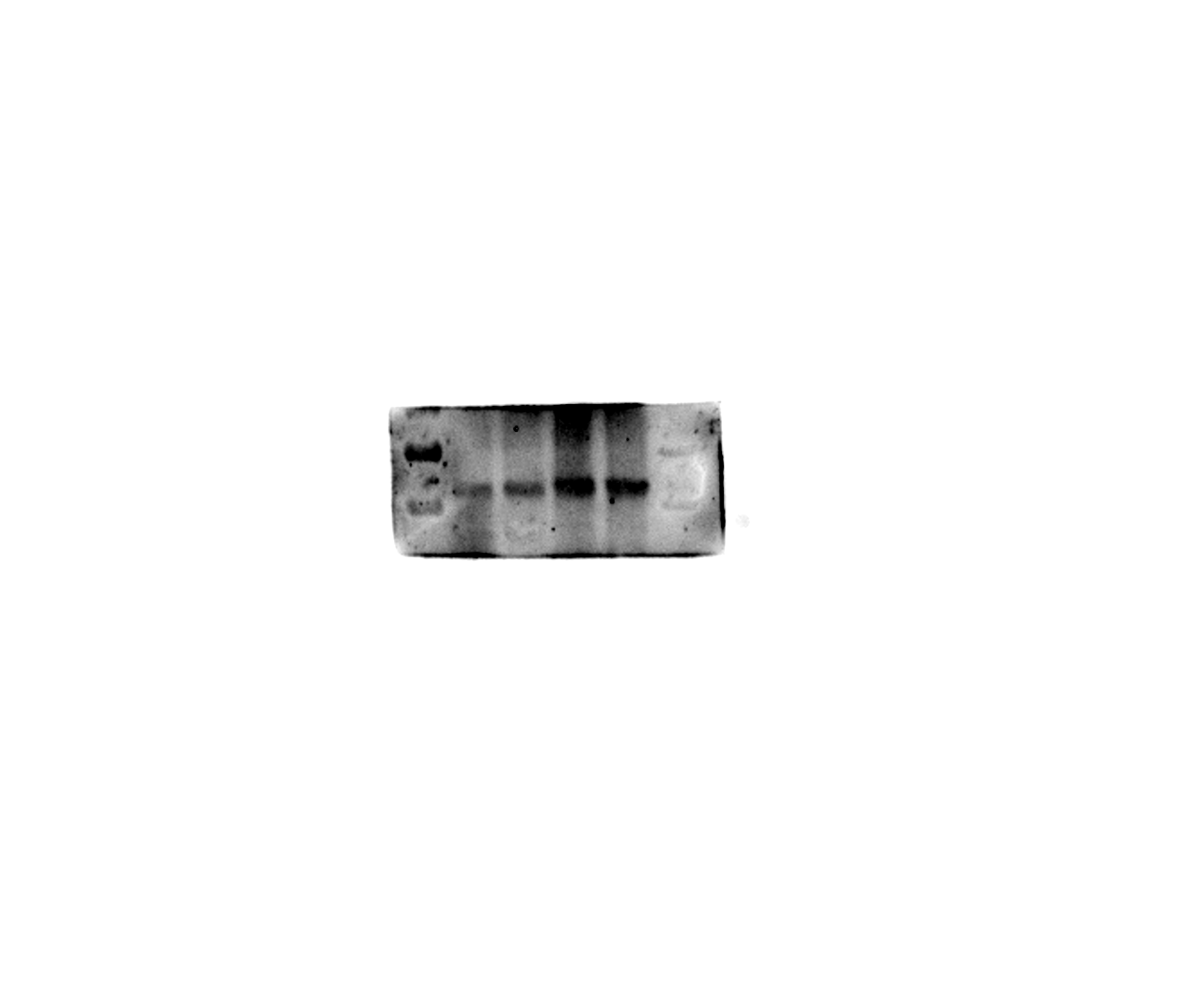


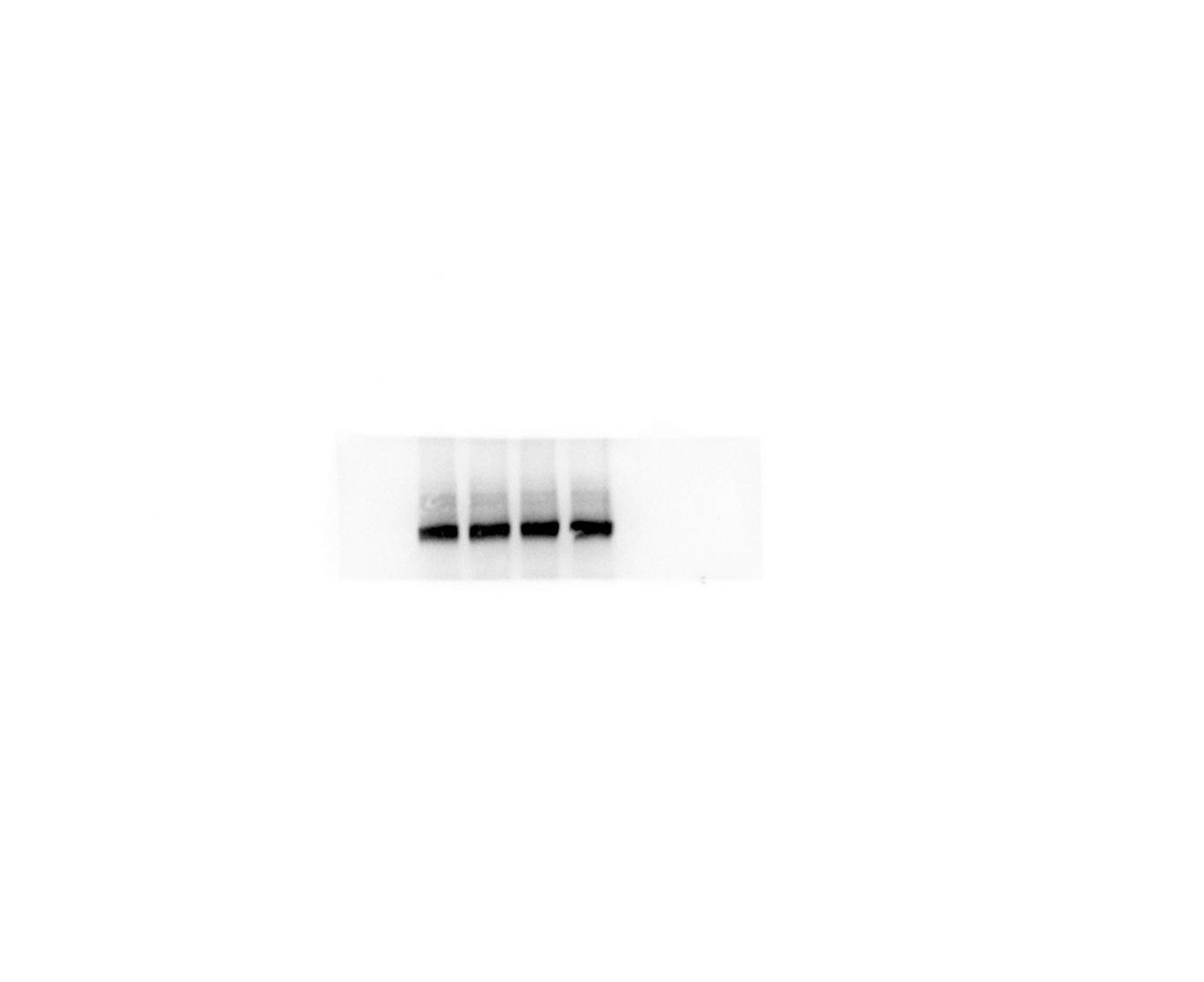


Figure 1J-STAT6

Figure 1J-ARG1


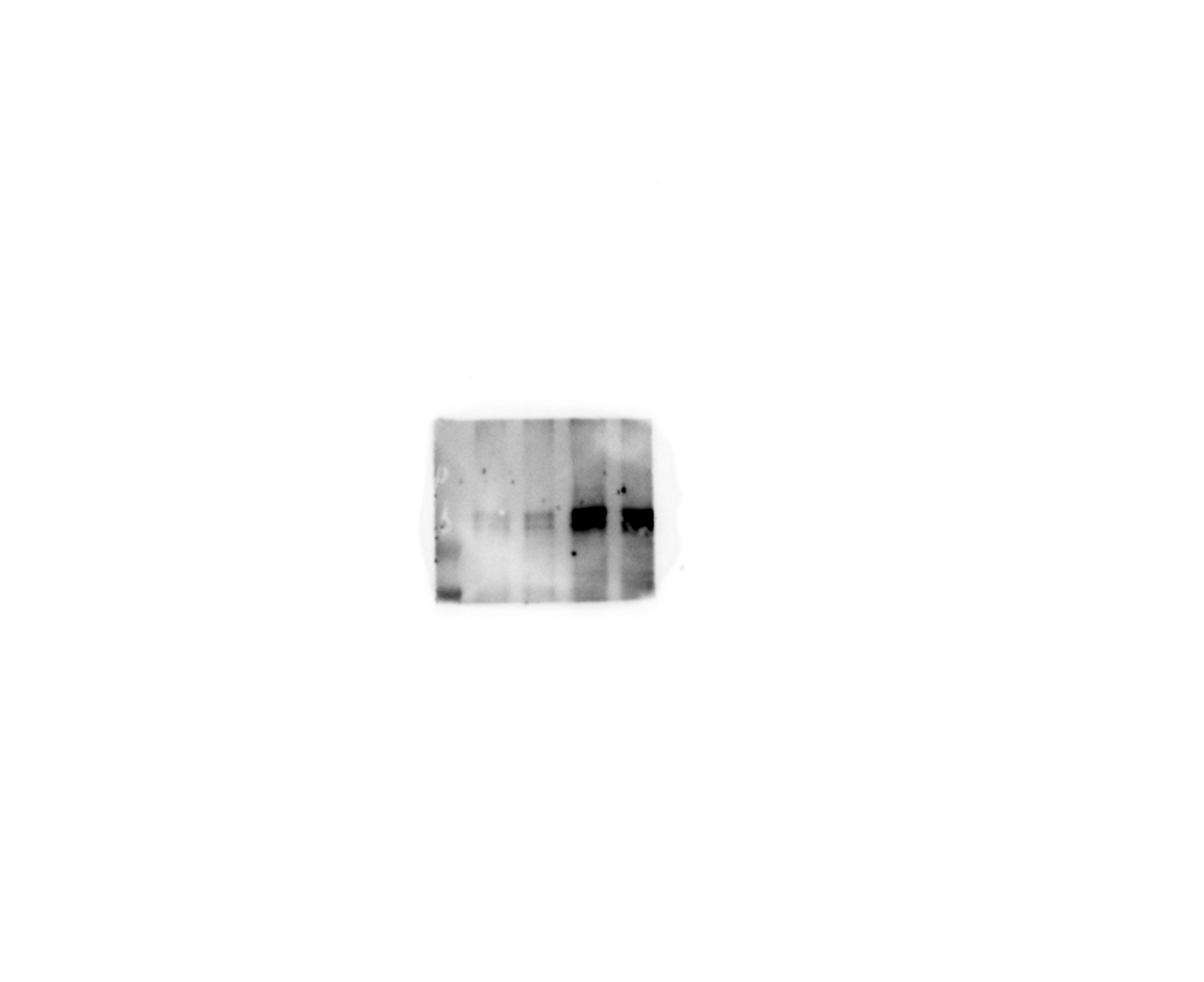

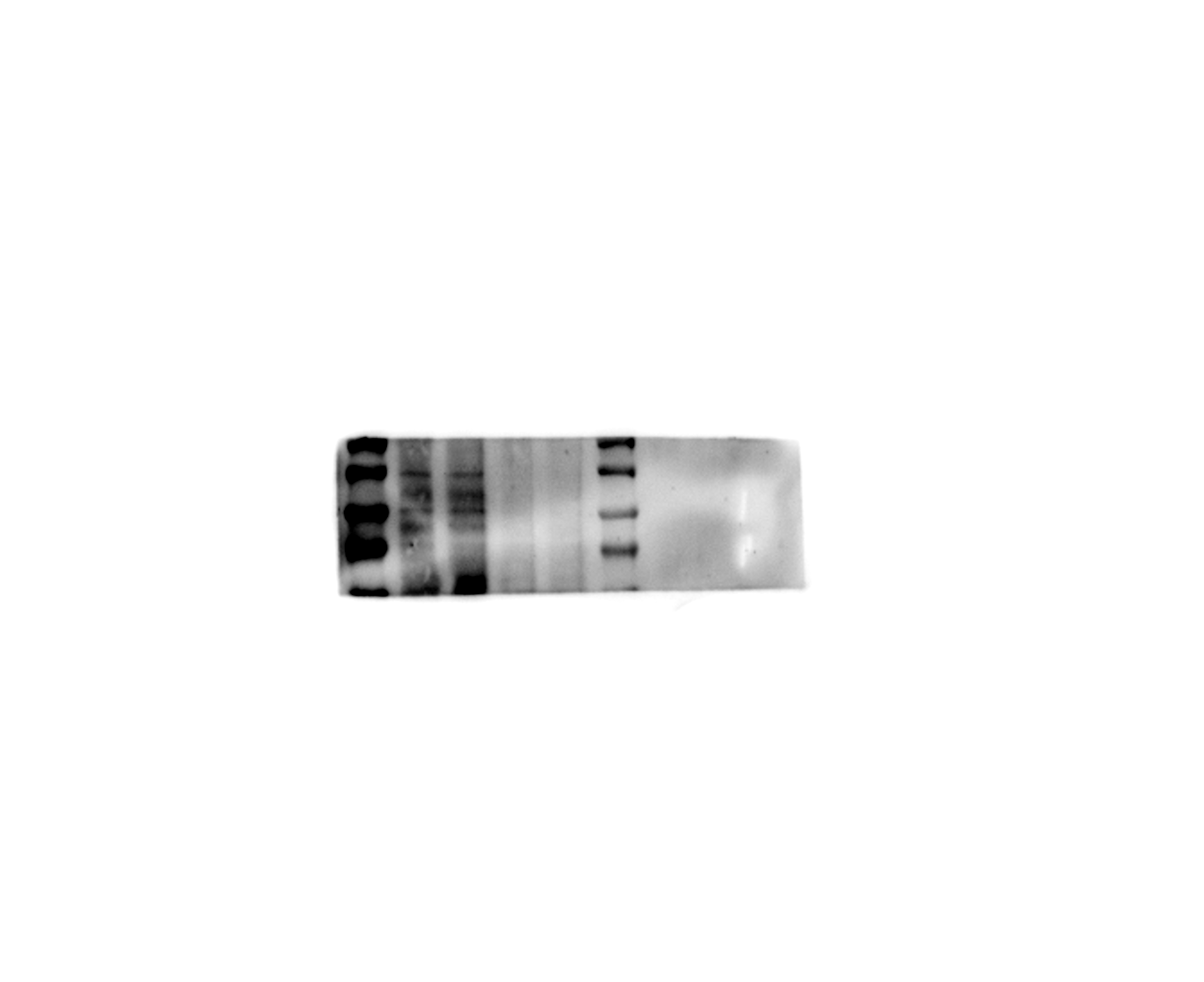

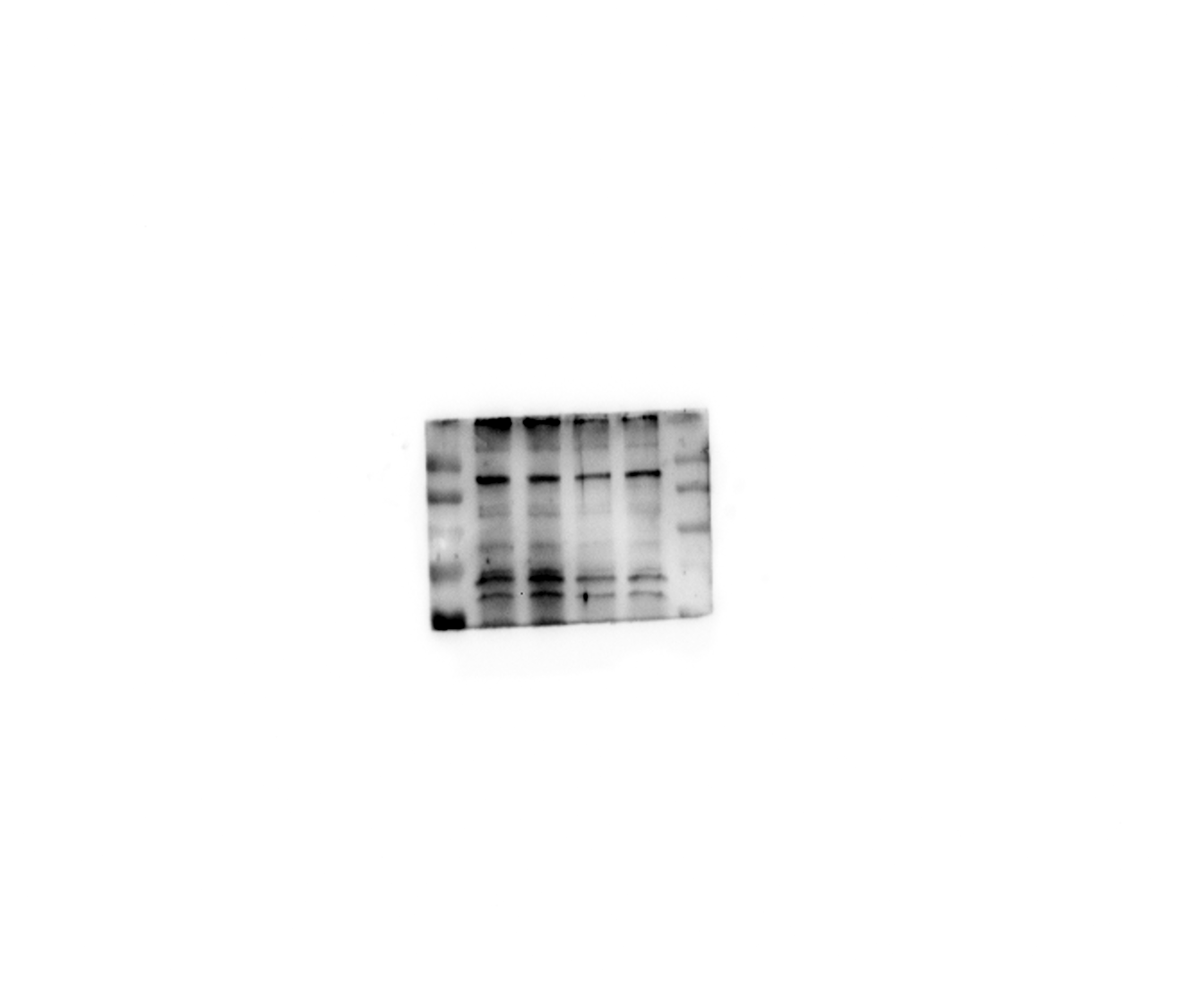


Figure 1J-p-STAT6

Figure 1J-p-STAT1

Figure 1J-INOS


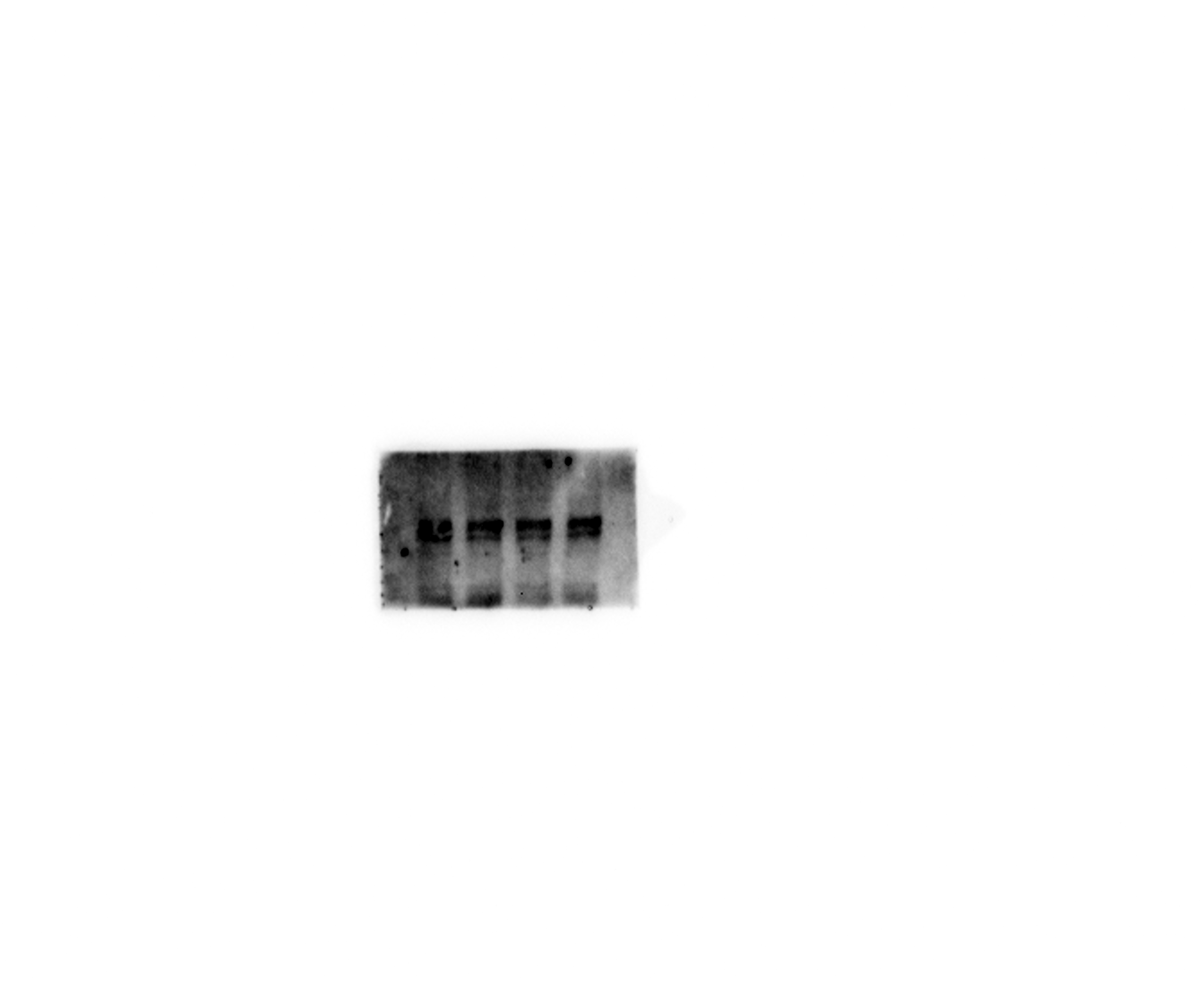


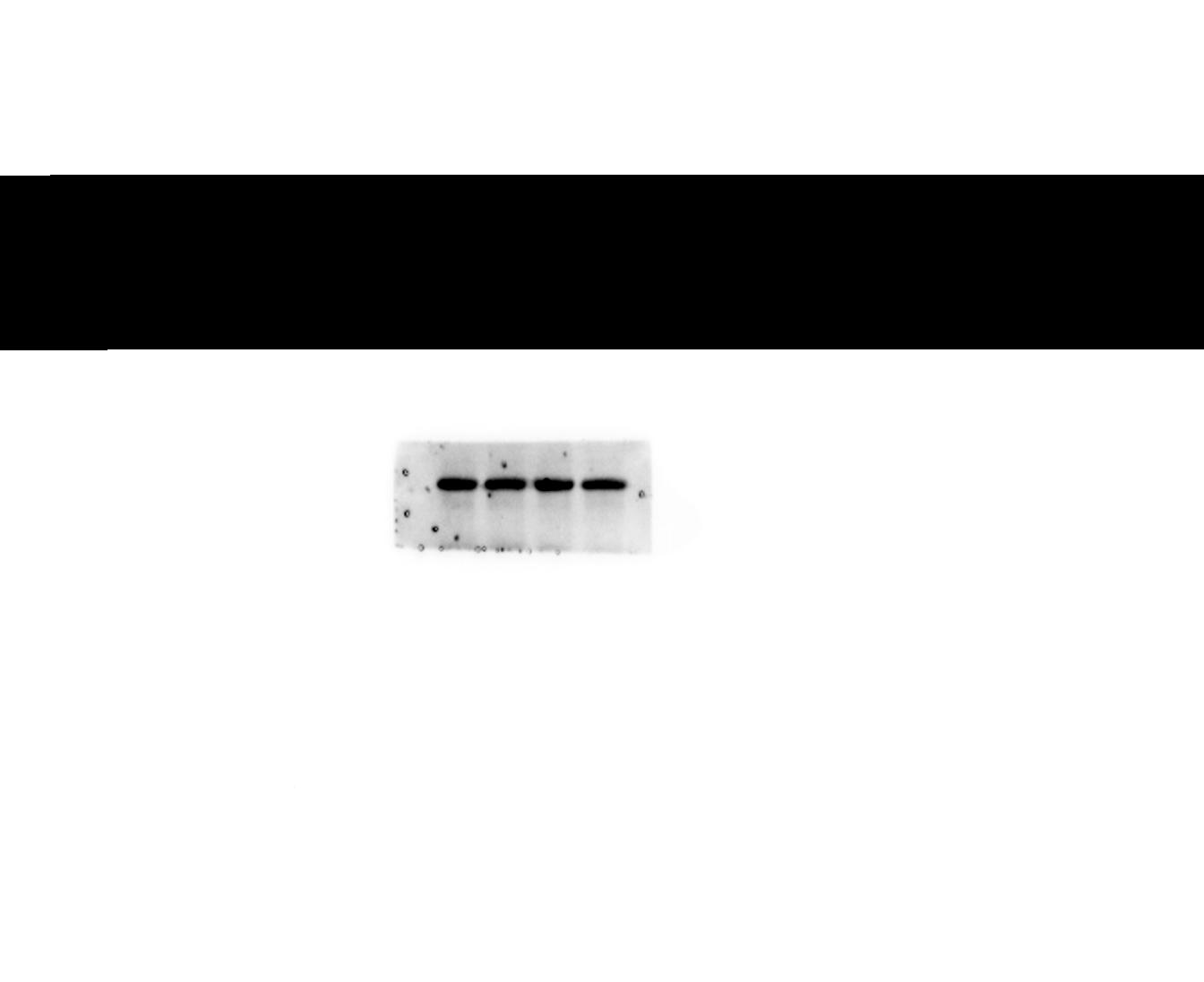


Figure 1J-Actin

Figure 1J-STAT1







Figure 3I-PINK1

Figure 3I-PGAM5







Figure 3I-Parkin

Figure 3I-pPPAR-γ







Figure 3I-PTEN

Figure 3I-FUNDC1







Figure 3K-pDRP1-616

Figure 3I-Actin




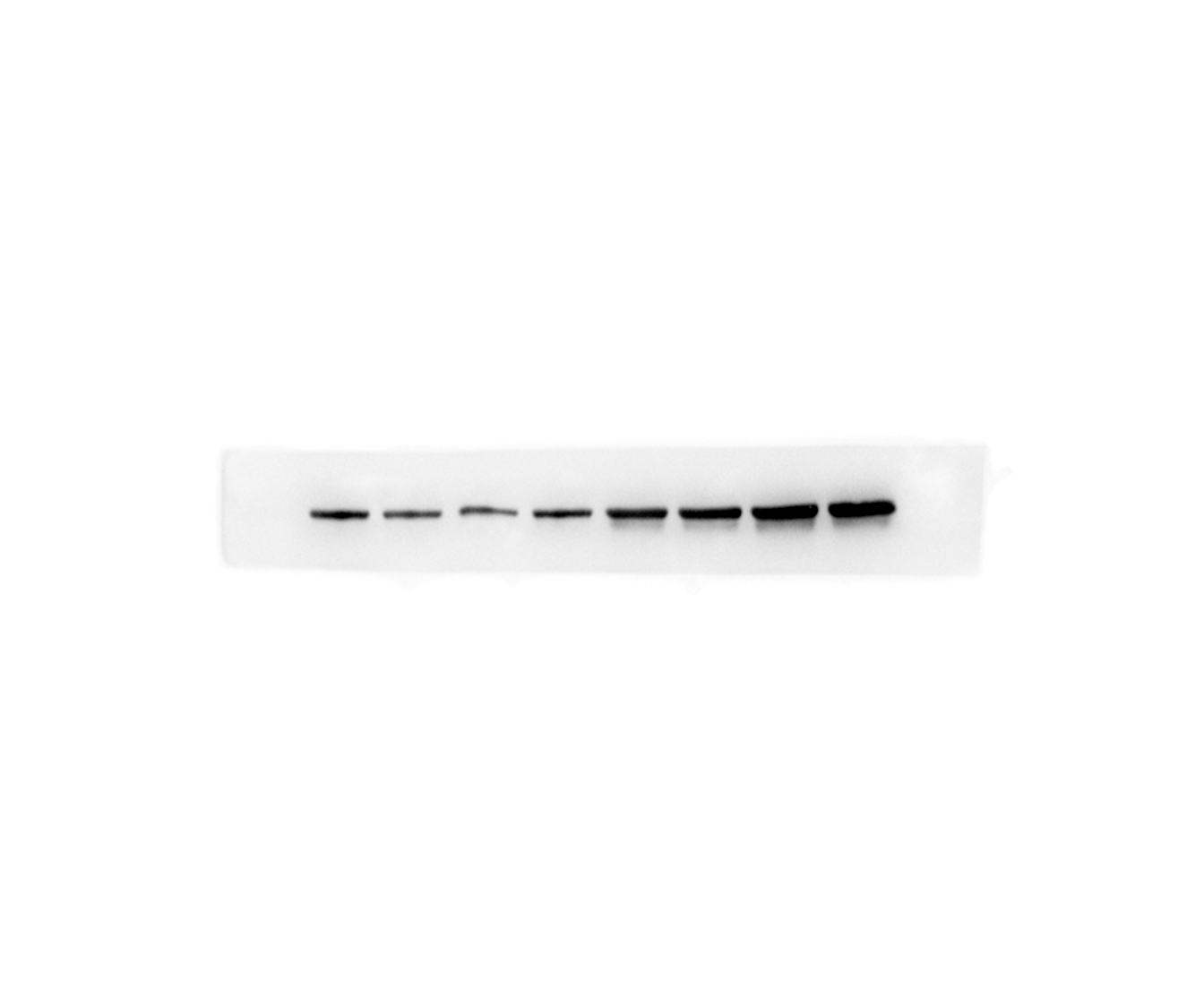


Figure 3K-RIPK3

Figure 3K-RIPK1


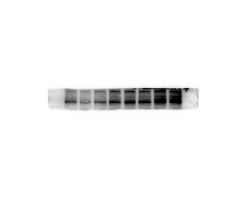

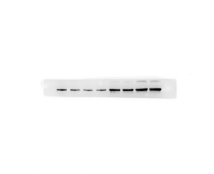







Figure 3K-DRP1

Figure 3K-pMLKL

Figure 3K-MLKL


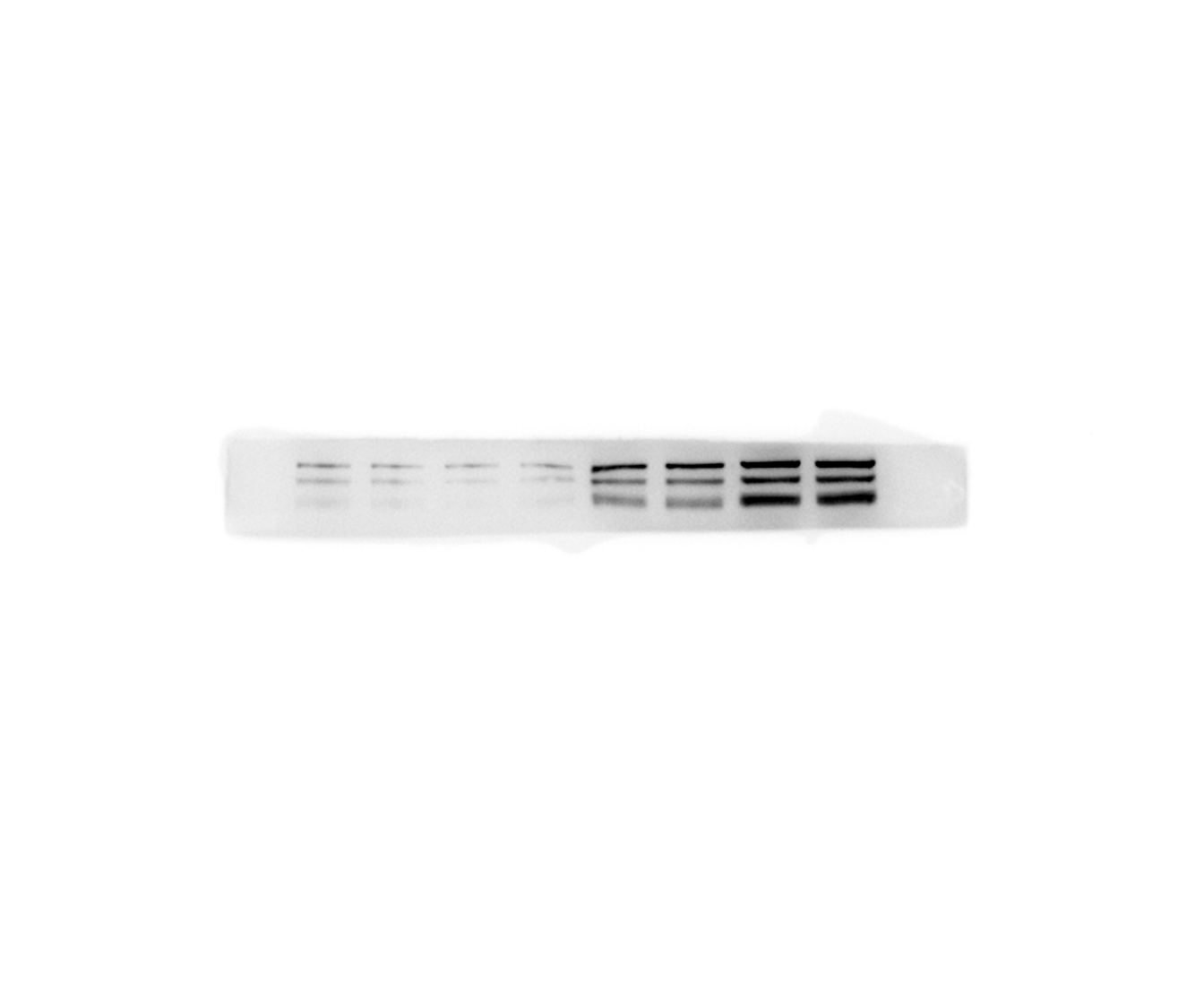


Figure 3K-Actin

Figure 3K-pDRP1


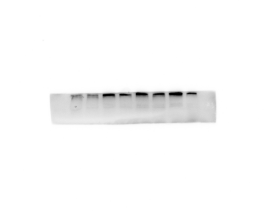

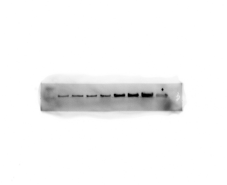

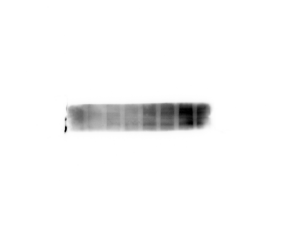


Figure 3M-pMLKL

Figure 3M-RIPK1

Figure 3M-RIPK3


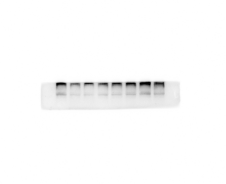

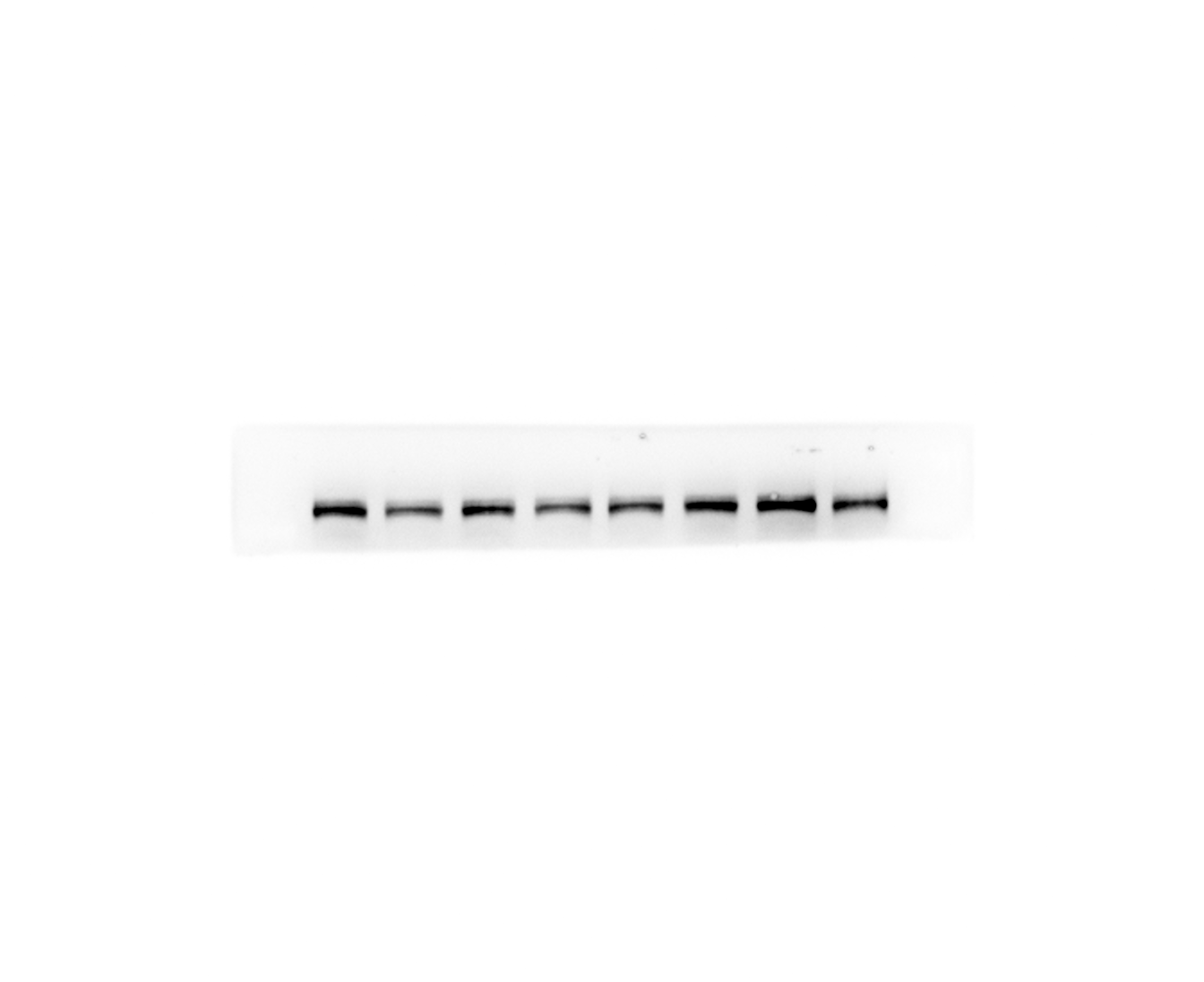





Figure 3M-MLKL

Figure 3M-DRP1

Figure 3M-pDRP1-s616


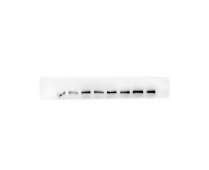


Figure 3M-Actin


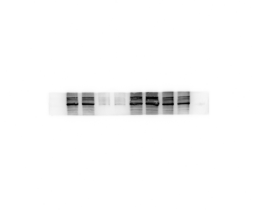

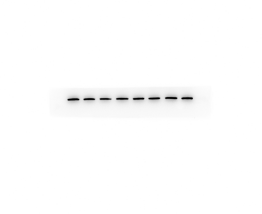

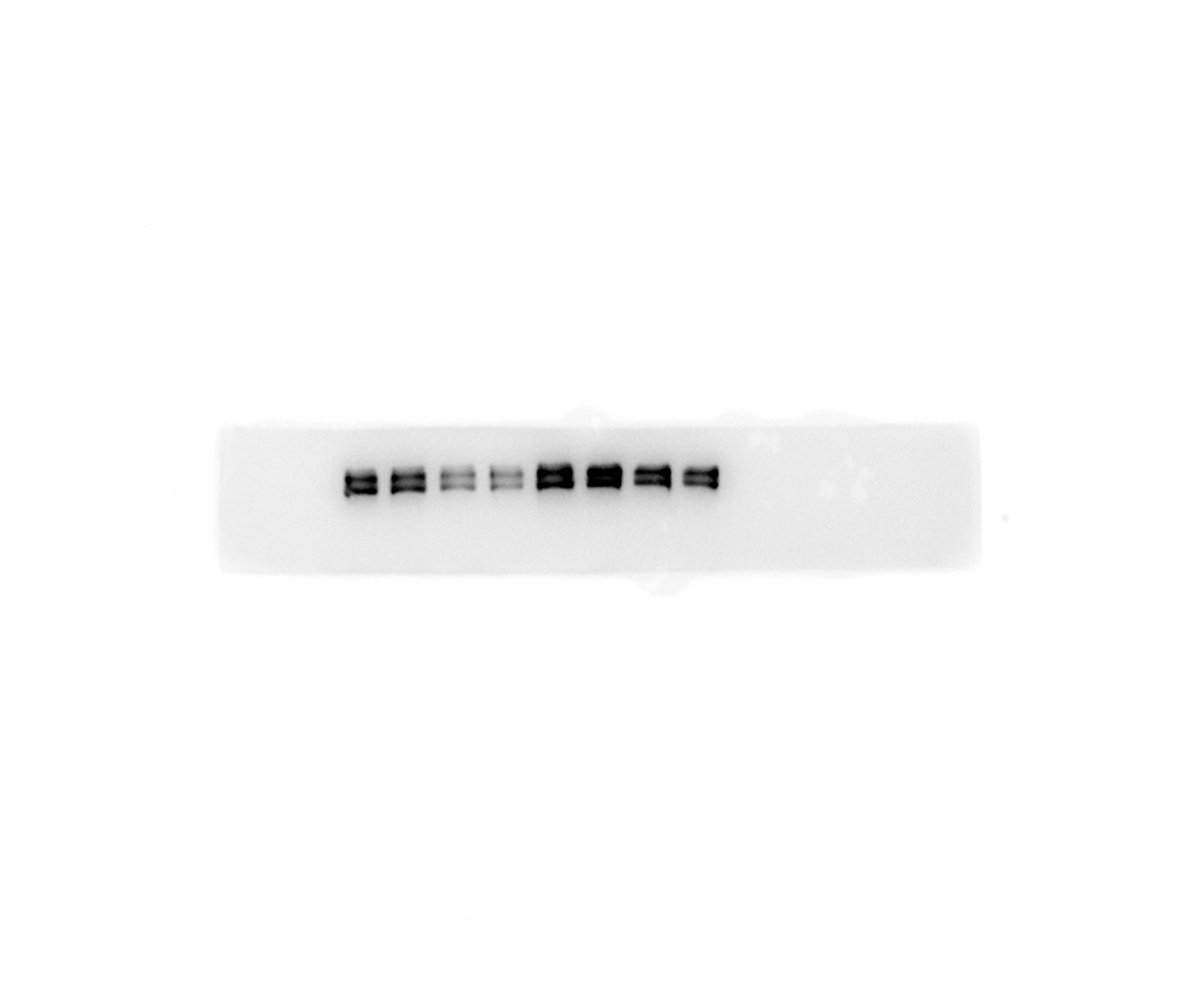


Figure 3N-CPT1a

Figure 3N-Actin

Figure 3N-ACC1


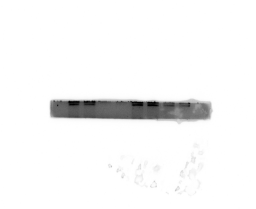

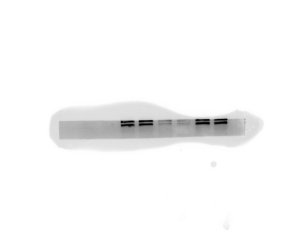

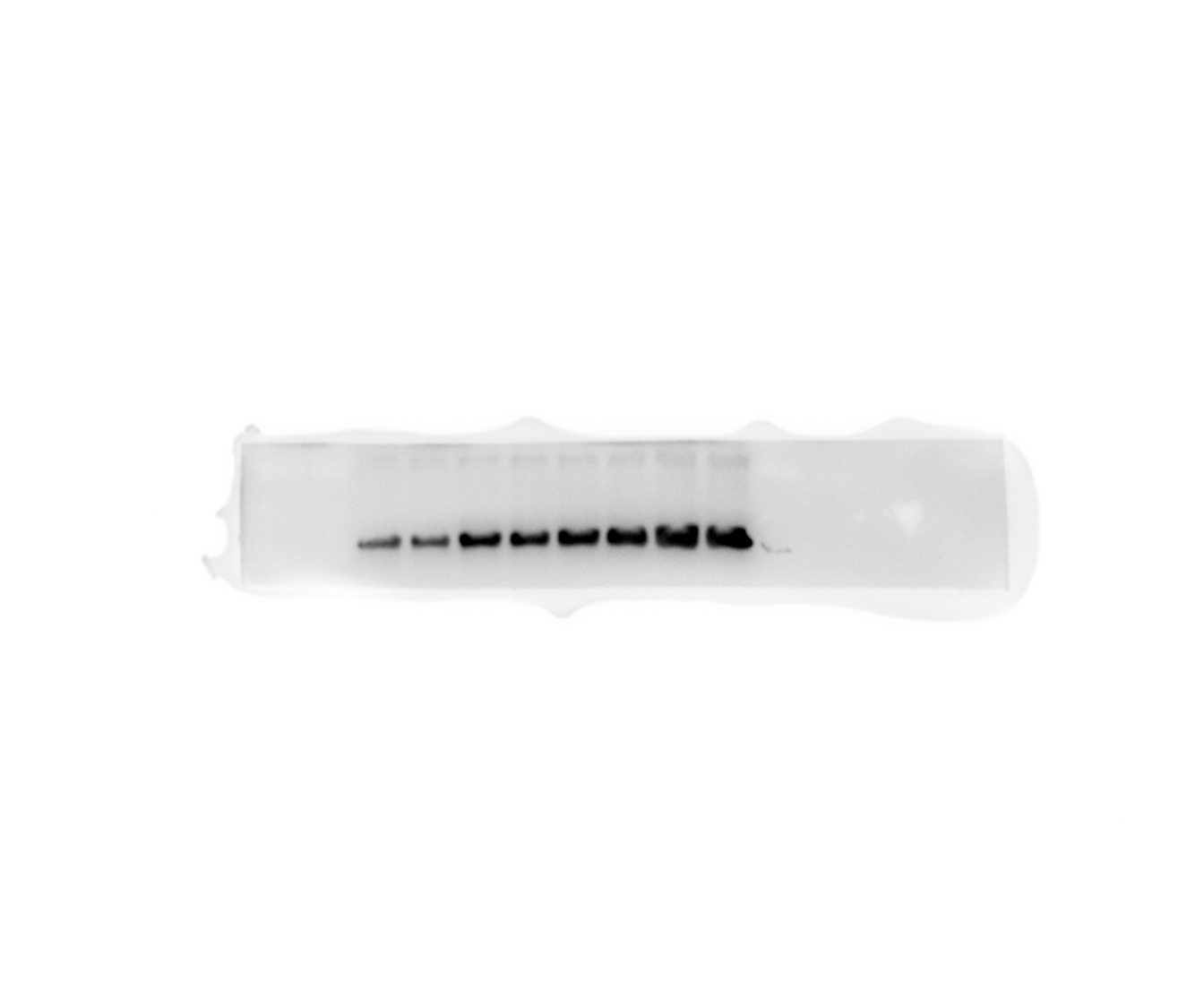


Figure 3N-HK2

Figure 3N-glut1

Figure 3N-pACC1


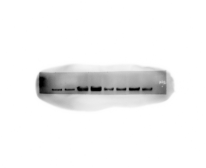

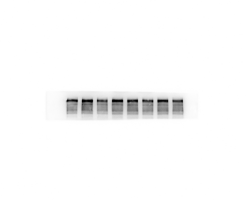

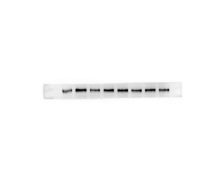


Figure 3P-pcMYC

Figure 3P-mTOR

Figure 3P-AMPK


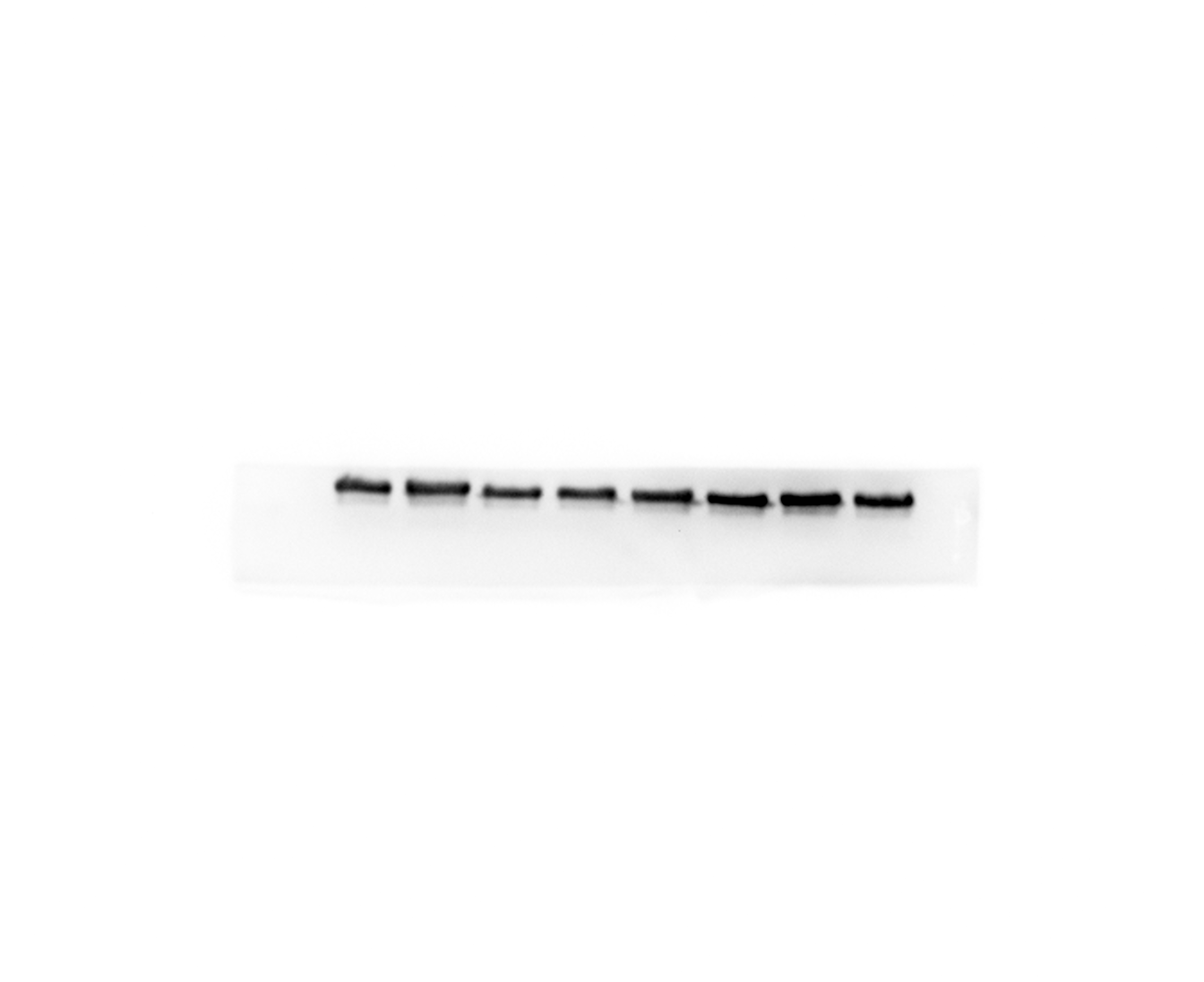

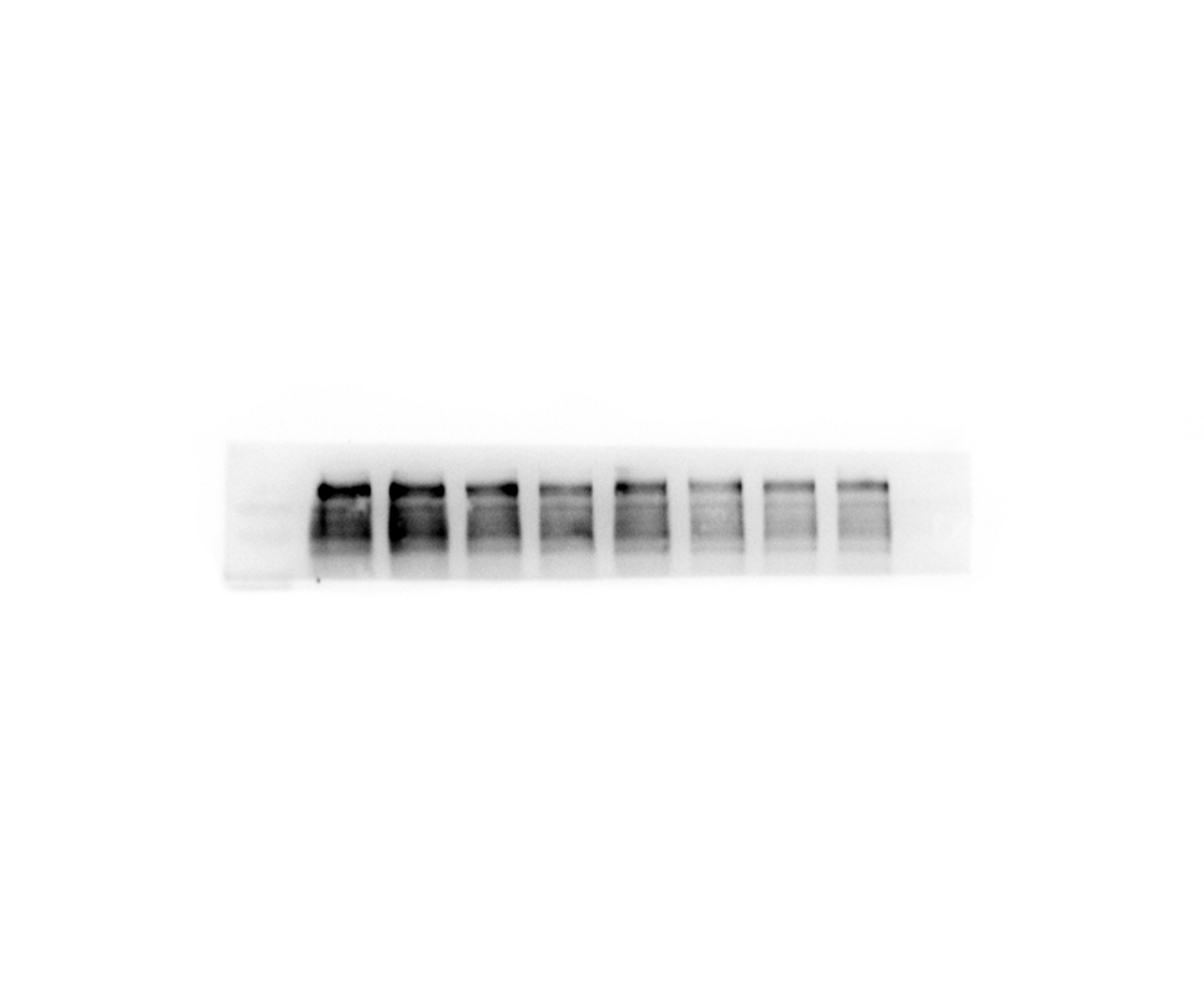


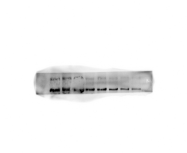


Figure 3P-p-AMPK

Figure 3P-Actin

Figure 3P-p-mtor


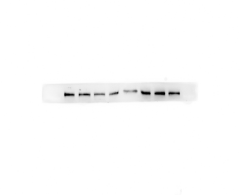


Figure 3P-cMYC









Figure 3Q-F4-pdrp1-616

Figure 3Q-F4-drp1

Figure 3Q-F4-Actin










Figure 3Q-F4-PTEN

Figure 3Q-F4-PGAM5

Figure 3Q-F4-pcmyc


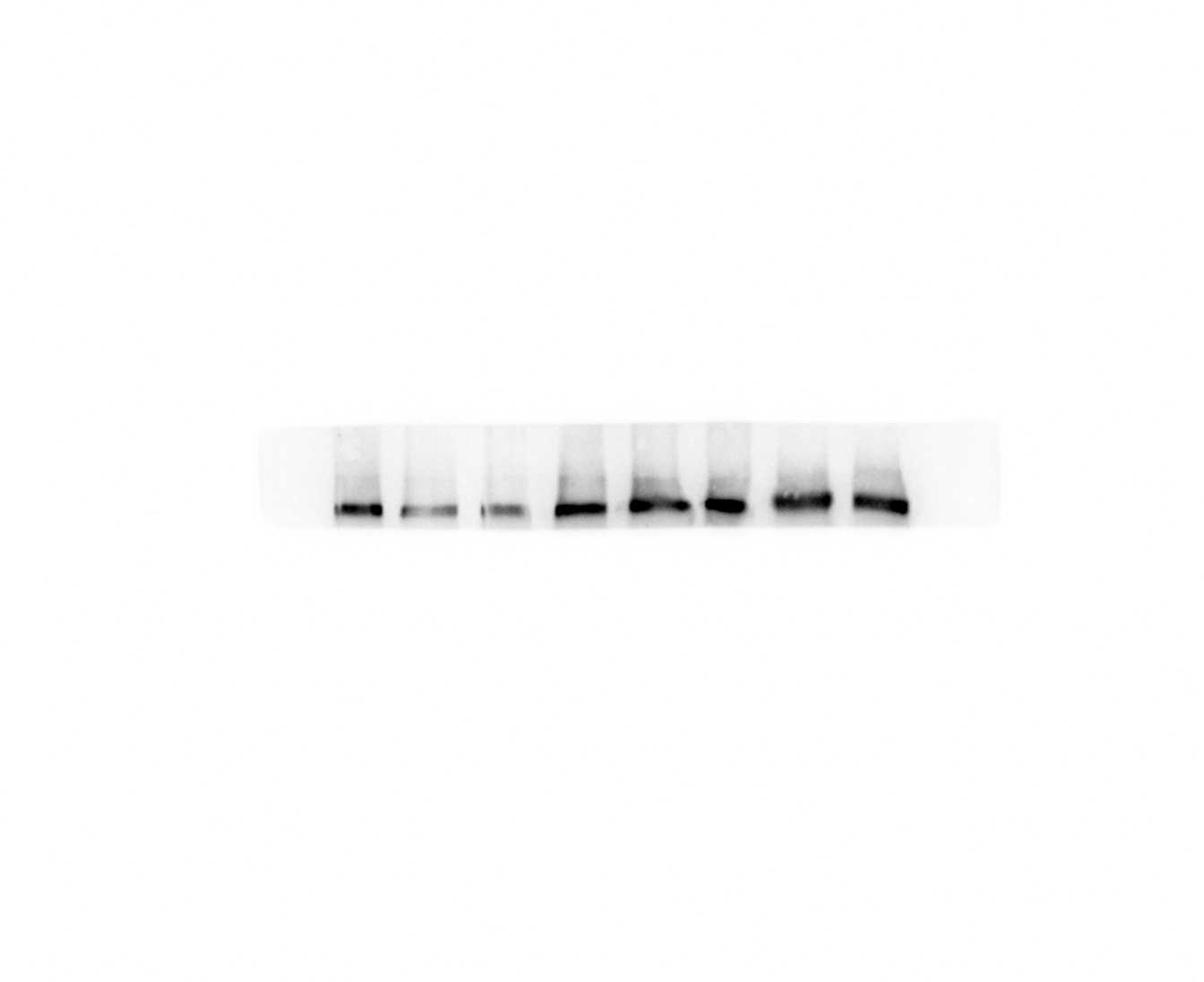


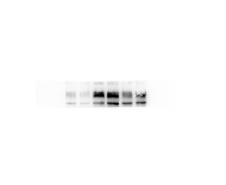


Figure 3Q-siRNA-cMYC

Figure 3Q-F4-cMYC


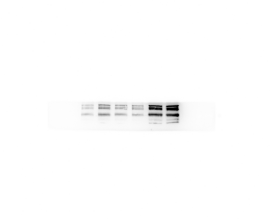




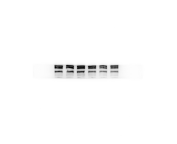


Figure 3Q-siRNA-pdrp1-616

Figure 3Q-siRNA-drp1

Figure 3Q-siRNA-Actin


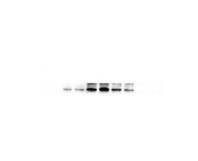

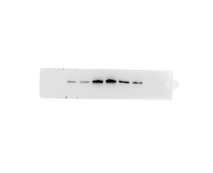

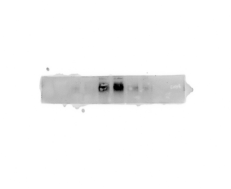


Figure 3Q-siRNA-pcmyc

Figure 3Q-siRNA-PGAM5

Figure 3Q-siRNA-PTEN








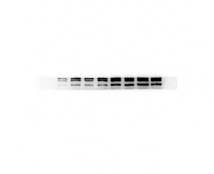


Figure 4C-RIPK1

Figure 4C-Actin

Figure 4C-bcl-2


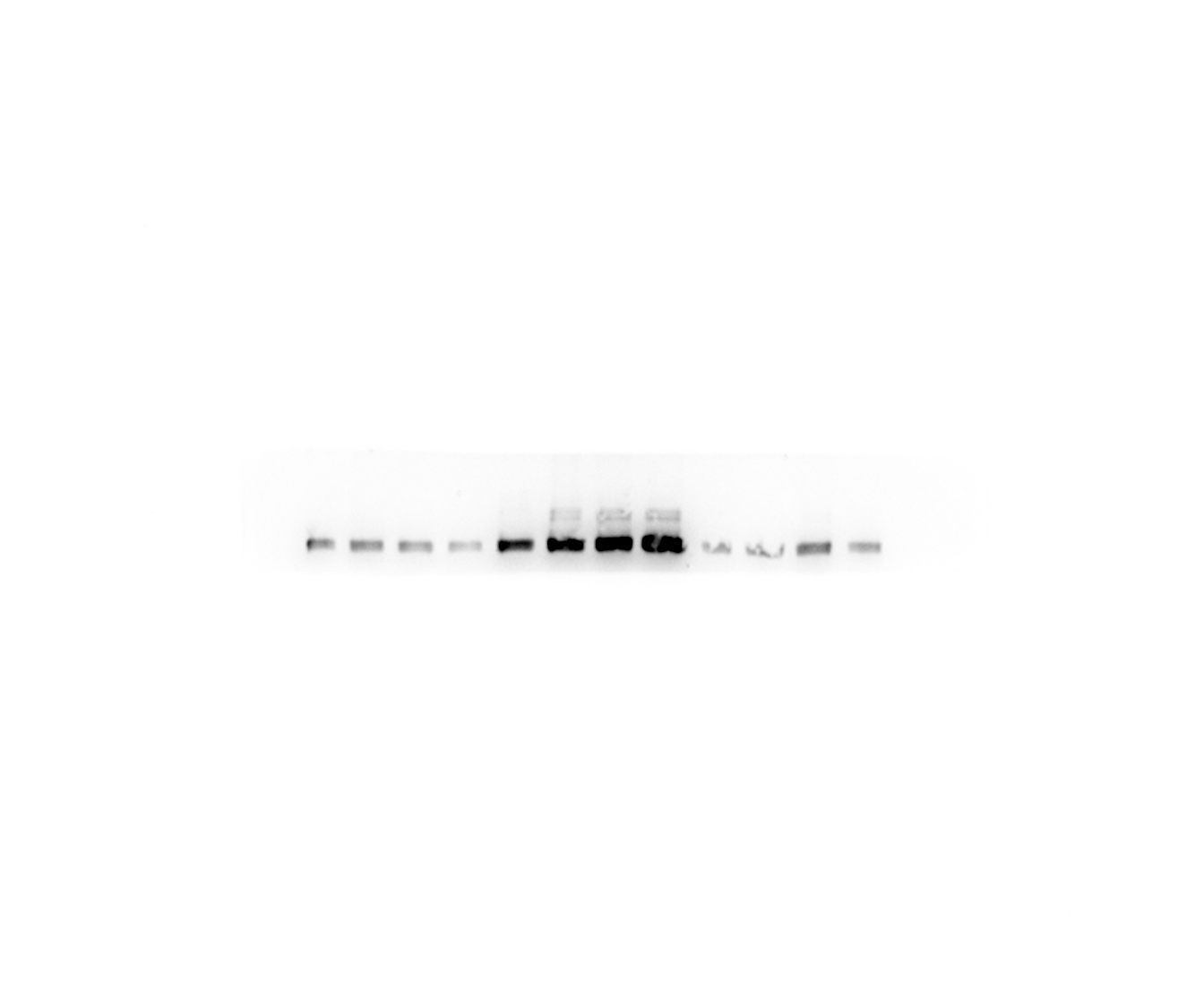


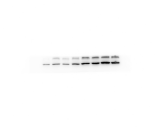


Figure 4C-MLKL









Figure 4C-DRP1

Figure 4C-DRP1

Figure 4C-RIPK3

Figure 4C-pMLKL

Figure 4C-pDRP1










Figure 4E-PINK1

Figure 4E-PGAM5

Figure 4E-Actin







Figure 4E-FUNDC1

Figure 4E-Parkin










Figure 4F-GSDMD

Figure 4F-caspase 1

Figure 4F-Actin







Figure 4F-NLRP3

Figure 4F-N-GSDMD









Figure 4G-PTEN

Figure 4G-p-mTOR

Figure 4G-mTOR










Figure 4G-Actin

Figure 4G-pcMYC

Figure 4G-cMYC











Figure 4I-DRP1

Figure 4I-pDRP1-616

Figure 4I-Actin







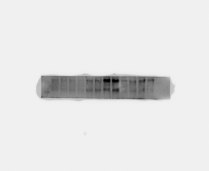


Figure 5D-ripk3

Figure 5D-p-drp1-616

Figure 5D-drp1


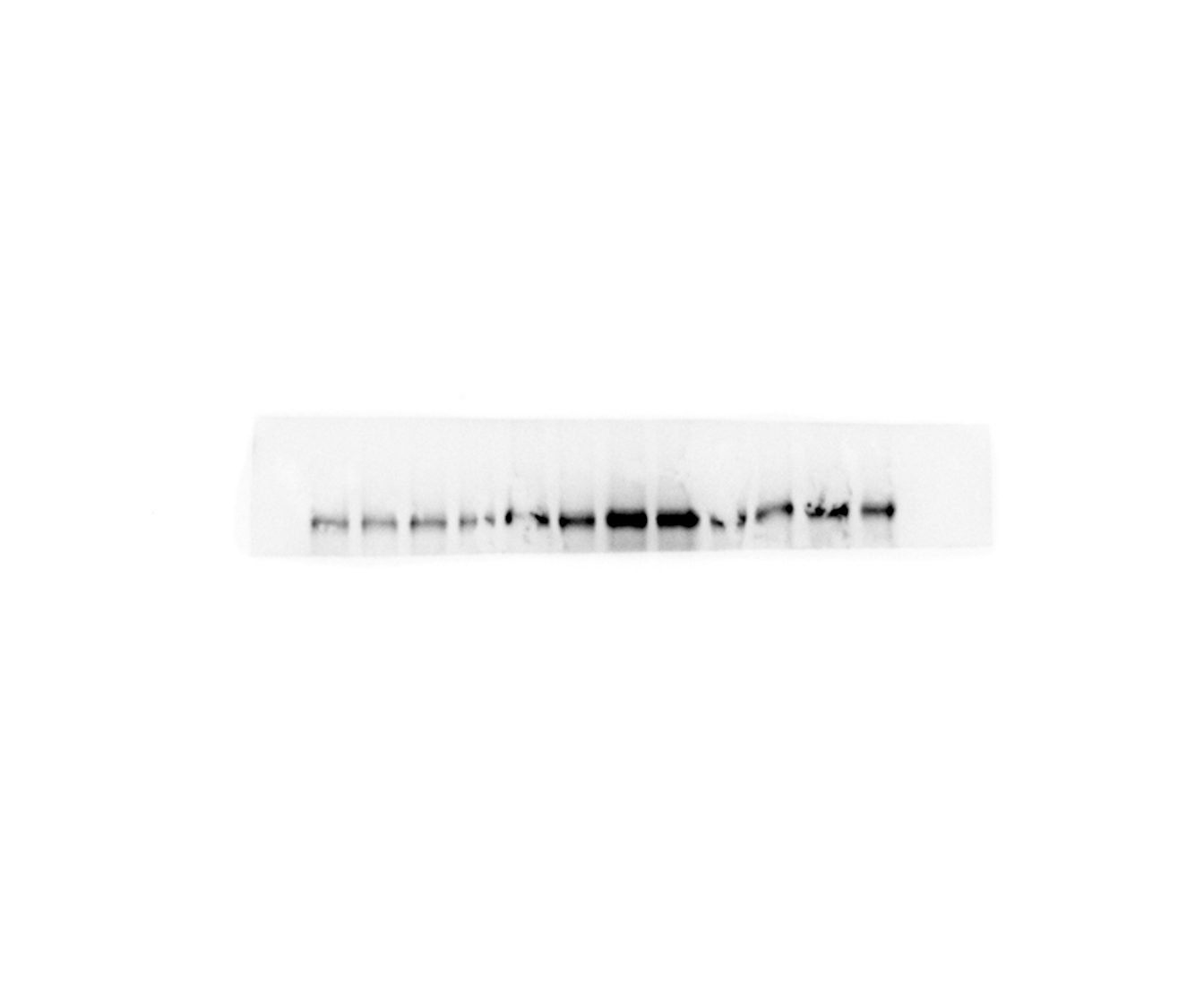

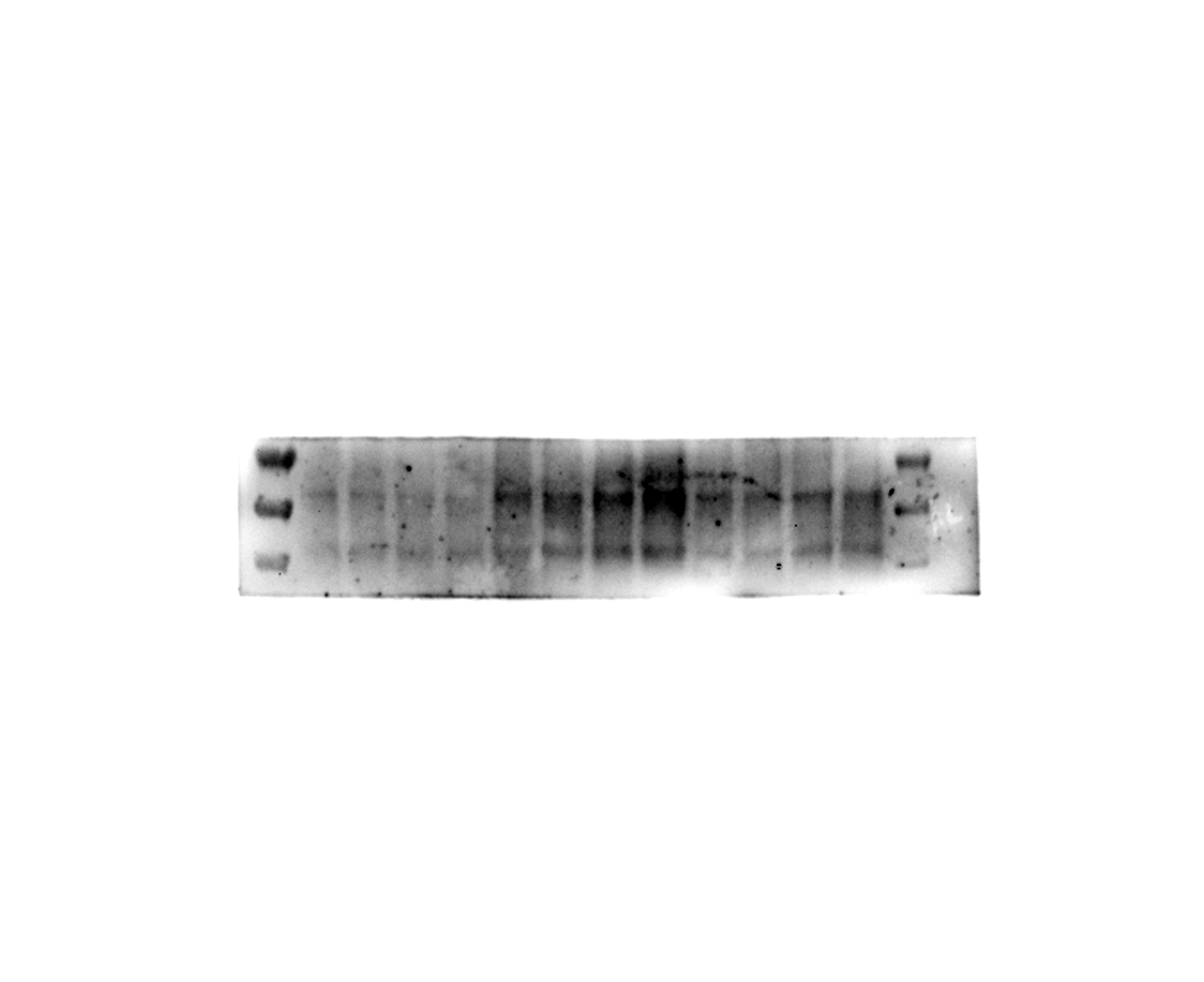





Figure 5D-p-mlkl

Figure 5D-mlkl

Figure 5D-ripk1








Figure 5D-Actin

Figure 5D-bcl-2








Figure 5E-Parkin

Figure 5E-PINK1

Figure 5E-PGAM5

Figure 5E-Actin

Figure 5E-FUNDC1

Figure 5E-PTEN

Figure 5F-akt

Figure 5E-LC3B

Figure 5F-p-akt

Figure 5F-NF-KB p65

Figure 5F-NF-KB p-p65

Figure 5F-p-c-myc

Figure 5F-c-myc

Figure 5F-HK2

Figure 5F-CPT1α

Figure 5F-Actin

Figure 5H-FUNDC1

Figure 5H-HK2

Figure 5H-cMYC

Figure 5H-PINK1

Figure 5H-P-MLKL

Figure 5H-PGAM5

Figure 5H-MLKL

Figure 6C-p-drp1-616

Figure 6C-drp1

Figure 6C-UCP1

Figure 6C-ripk3

Figure 6C-ripk1

Figure 6C-p-mlkl

Figure 6C-mlkl

Figure 6C-Actin

Figure 6D-Parkin

Figure 6D-PINK1

Figure 6D-PGAM5

Figure 6D-Actin

Figure 6D-bcl2

Figure 6D-FUNDC1

Figure 6J-p-STAT6

Figure 6J-ARG1

Figure 6J-p-STAT1

Figure 6J-Actin

Figure 6J-STAT1

Figure 6J-STAT1

Figure 6J-INOS

Figure 6J-STAT6

Figure 7A-p38

Figure 7A-PTEN

Figure 7A-p-pparγ

Figure 7A-UCP1

Figure 7A-ATF2

Figure 7A-p-p38

Figure 7A-Actin

Figure 7B-P38

Figure 7B-PTEN

Figure 7B-p-pparγ

Figure 7B-P-P38

Figure 7B-UCP1

Figure 7B-ATF2

Figure 7C-P38

Figure 7B-Actin

Figure 7C-p-p38

Figure 7C-PTEN

Figure 7C-p-pparγ

Figure 7C-Actin

Figure 7C-UCP1

Figure 7C-ATF2

Figure 7F-PGAM5

Figure 7F-p-c-myc

Figure 7F-UCP1

Figure 7F-ripk1

Figure 7F-p-drp1-616

Figure 7F-PINK1

Figure 7F-Actin

Figure 7F-p-mlkl

Figure 7F-p-ripk3

Figure 7F-ripk3

Figure 7F-p-ripk3

Figure 7F-cMYC

Figure 7F-cMYC

Figure 7F-cMYC

Figure 7F-cMYC

Figure 7F-DRP1

Sup Fig3B-NRF2

Sup Fig3B-Actin

Sup Fig3B-Actin

Sup Fig3B-NRF1

Sup Fig3B-TFAM

Sup Fig3B-TFAM

Sup Fig3B-TFAM

Sup Fig4C-Ferritin

Sup Fig4C-Actin

Sup Fig4C-ALOX12

Sup Fig4C-GPX4
